# Serotonergic neuron ribosomal proteins regulate the neuroendocrine control of *Drosophila* development

**DOI:** 10.1371/journal.pgen.1010371

**Published:** 2022-09-01

**Authors:** Lisa Patricia Deliu, Michael Turingan, Deeshpaul Jadir, Byoungchun Lee, Abhishek Ghosh, Savraj Singh Grewal

**Affiliations:** Clark H Smith Brain Tumour Centre, Arnie Charbonneau Cancer Institute, Alberta Children’s Hospital Research Institute, and Department of Biochemistry and Molecular Biology Calgary, University of Calgary, Alberta, Canada; University of Virginia, UNITED STATES

## Abstract

The regulation of ribosome function is a conserved mechanism of growth control. While studies in single cell systems have defined how ribosomes contribute to cell growth, the mechanisms that link ribosome function to organismal growth are less clear. Here we explore this issue using *Drosophila Minutes*, a class of heterozygous mutants for ribosomal proteins. These animals exhibit a delay in larval development caused by decreased production of the steroid hormone ecdysone, the main regulator of larval maturation. We found that this developmental delay is not caused by decreases in either global ribosome numbers or translation rates. Instead, we show that they are due in part to loss of Rp function specifically in a subset of serotonin (5-HT) neurons that innervate the prothoracic gland to control ecdysone production. We find that these effects do not occur due to altered protein synthesis or proteostasis, but that *Minute* animals have reduced expression of synaptotagmin, a synaptic vesicle protein, and that the *Minute* developmental delay can be partially reversed by overexpression of synaptic vesicle proteins in 5-HTergic cells. These results identify a 5-HT cell-specific role for ribosomal function in the neuroendocrine control of animal growth and development.

## Author summary

Ribosomes are essential cellular organelles required for production of new proteins. Extensive studies have shown how ribosome synthesis and function are important for growth at the cellular level, but less is known about how these processes control tissue and body growth. We have explored this issue by studying a class of Drosophila (fruit fly) mutants–the *Minutes*–which lack one functional copy of a ribosomal protein gene and, as a result, show a slowed rate of development from embryo to adulthood. An important phase in the life cycle of animals is the transition from the juvenile, growth stage to sexually mature adults. In fruit flies, this occurs as larvae mature into pupae and is controlled by a pulse of the steroid hormone ecdysone, which is secreted from an endocrine organ called the prothoracic gland (PG). We find that *Minute* animals have disrupted production of ecdysone and we pinpoint this defect to loss of ribosomal protein function within a subset of neurons that project from the brain to the PG and that control ecdysone production through the neurotransmitter, serotonin. Our results highlight how ribosomal protein function in a subset of neurons can control overall body growth and development.

## Introduction

The regulation of ribosome and protein synthesis are conserved mechanisms of growth control. Several decades of studies in unicellular systems such as *E*. *coli*, yeast and cultured mammalian cells have defined both the signaling pathways that couple growth cues to ribosome synthesis and function, and the mechanisms by which changes in mRNA translation drive cell growth and proliferation [1–4]. However, the mechanisms that operate in whole animals during developmental growth are less clear. In these contexts, body growth is not determined solely by processes that govern cell-autonomous growth, but also by inter-organ communication to ensure coordinated growth and development across all tissues and organs [5–7]. Hence, tissue specific changes in ribosome function have the potential to mediate non-autonomous effects on whole-body physiology to control organismal development.

The complex links between ribosome function and animal development are exemplified by the organismal biology of ribosomal proteins (Rps) [8,9]. Metazoan ribosomes have 70–80 Rps, and mutants for almost all of these are homozygous lethal in animals, emphasising their essential role in ribosome synthesis and function. However, in many cases Rp mutants show dominant phenotypes as heterozygotes. These phenotypes are often specific to the affected Rp and can give rise to tissue-specific effects that cannot be explained simply by lowered overall protein synthesis and growth rates. For example, in zebrafish certain *rp/+* mutants can develop peripheral nerve tumors [10]. Similarly, some *Drosophila* Rp mutants develop selective tissue overgrowth phenotypes [11,12]. Several *Rp/+* mutants in mice have also been shown to each exhibit tissue specific developmental defects that differ based on the Rp affected. For example, *rpl38/+* mice show specific skeletal segmentation defects [13], *rps14/+* mice show defects in blood development [14], and *rpl27a/+ mice* show defects in cerebellar development [15]. The dominant effects of *rp/+* mutations also extend to humans, where several pathologies, collectively termed ribosomopathies, are caused by heterozygosity for Rp mutations, and lead to tissue-specific effects such as blood disorders, congenital growth defects, and predisposition to cancer [16–18]. The mechanisms that determine these dominant effects of *rp/+* mutations are not fully clear but are thought to involve selective alterations in mRNA translation. These alterations may occur either as a result of lowered ribosome numbers or due to ribosome heterogeneity, where ribosomes with different complements of Rps have been proposed to have different translational properties [19–23]. These studies emphasise the importance of further work to understand how Rp function contributes to organismal growth and development.

*Drosophila* larvae have provided an excellent model system in which to define the cell-, tissue- and body-level mechanisms that control developmental growth [6,24,25]. Larvae grow almost 200-fold in mass over 4–5 days before undergoing metamorphosis to the pupal stage. This developmental transition is controlled by a pulse of secretion of the steroid hormone, ecdysone, from the prothoracic gland (PG), which then acts on tissues to stimulate pupation at the end of the larval period [26–28]. The timing of this pulse is under control of two separate subsets of neurons expressing either the neuropeptide, PTTH, or the neuromodulator serotonin (5-HT), that each innervate the PG and stimulate ecdysone production [29–31]. This neuroendocrine network integrates signals from the environment and other tissues to ensure proper timing of the ecdysone pulse and the larval-pupal transition. For example, nutrient signals can act on both the 5-HT neurons and the PG to ensure proper coupling of development maturation with nutrients [31,32]. Epithelial disc damage also leads to a delay in larval development to allow time for proper tissue regeneration before transition to the pupal stage. One way that this delay is mediated is by suppression of PTTH signaling by dilp8, an insulin/relaxin-like peptide that signals from damaged discs to a subset of Lgr3 receptor expressing neurons that inhibit PTTH neuronal activity [33–37]. In addition, the inflammatory cytokine, Upd3 can signal directly from damaged discs to the PG to suppress ecdysone and delay development [38].

An interesting class of mutants that exhibit alterations in larval development are the *Minutes* [39,40]. These are dominant mutants that are classically described by their developmental delay and short bristles. Almost all *Minutes* are *rp/+* mutants and they have perhaps been best studied in context of cell competition, a process in which mosaic clones of *rp/+* cells in imaginal disc epithelia are outcompeted and killed by surrounding wild-type (*+/+*) cells. Several mechanisms have been described to account why *rp/+* cells are outcompeted including altered proteostasis [41,42], competition for *dpp* growth factor [43], induction of innate immune signaling [44,45], and induction of the transcription factor Xrp1 [46,47]. Given that *rp* genes are spread across the genome, cell competition may be a surveillance process to eliminate aneuploid cells, as marked by reduced *rp* gene copy number, to ensure proper tissue growth and homeostasis [48].

Although much has been learned about cell competition, less is known about why *rp/+* show a delay in development. Interestingly, some recent studies of the disc-intrinsic, mechanisms of *rp/+* cell competition effects can also partially account for the organismal delay in development. For example, disc-specific Rp knockdown stimulates Xrp1 induction of dilp8 [49], and both loss of Xrp1 and disc-specific knockdown of dilp8 can each partially reverse the delay in development seen in *rp/+* animals [47,50,51]. Loss of Rp function specifically in the PG can also explain the overall delay in development in *rps6/+* animals [52]. These results suggest the overall delay in organismal development seen in *rp/+* animals may result from specific tissue non-autonomous effects of Rps. However, the extent of these non-autonomous effects is not fully clear.

Here we provide further evidence for tissue-specific effects of Rp in the control of larval development. We describe how loss of Rp function specifically in 5-HT neurons that innervate the PG can, in part, explain the developmental delay in *Minute* animals.

## Results

### Rps13/+ animals do not show a global decrease in ribosome levels or protein synthesis

**Fig 1:**
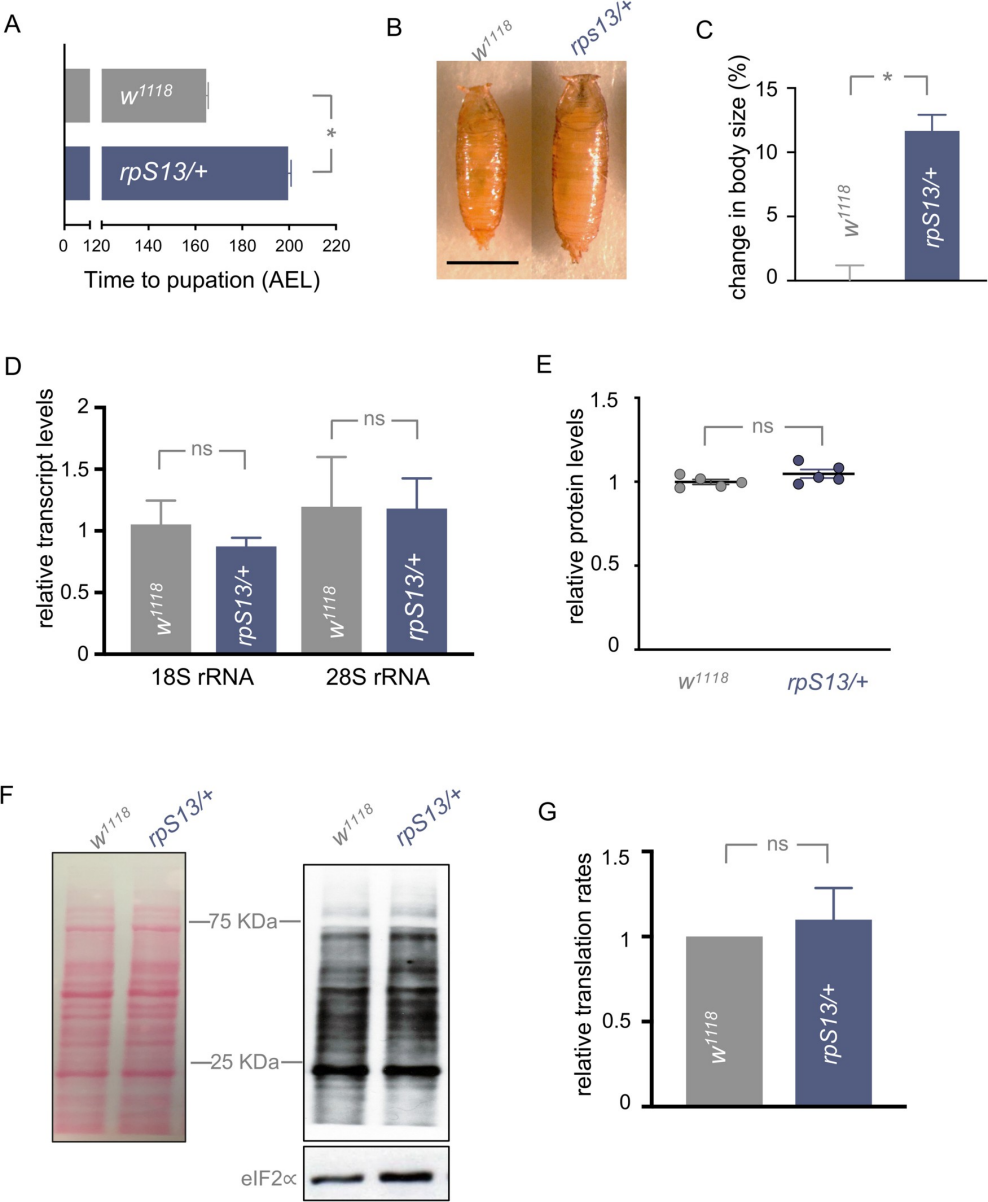
Developmental delay of *rpS13/+* animals is not due to a decrease in ribosome numbers or reduced translation rates. (A) Developmental timing from larval hatching to pupation of *w*^*1118*^ and *rpS13/+* animals, n = 147 and 170 respectively. Data are presented as +/- SEM. *p < 0.05, Mann-Whitney U test. (B) Representative images of *w*^*1118*^ and *rpS13/+* pupae. Scale bar, 1000 μm. (C) Pupal volume of *rpS13/+* (n = 212) and *w*^*1118*^ controls (n = 229). Data are presented as +/- SEM. *p < 0.05, Mann-Whitney U test. (D) Transcript levels of 18S and 28S rRNA in whole-body RNA samples from *w*^*1118*^ and *rpsS13/+* third instar wandering larvae. n = 4 independent samples per genotype. Data are presented as +/- SEM. ns = not significant, Student’s t-test. (E) Relative protein concentration levels from third instar wandering *w*^*1118*^ and *rpsS13/+* larvae. Absorbance was measured at 465nm using the Bradford assay, n = 5 independent samples per genotype. Individual data points are plotted, and the bars represent mean +/- SEM. ns = not significant, Student’s t-test. (F) Whole-body puromycin labelling of *w*^*1118*^ and *rpsS13/+* third instar wandering larvae. Left, Ponceau S staining showing total protein. Right, anti—puromycin and anti—eIF2∝ (loading control) immunoblots. (G) Quantification of puromycin staining, n = 6 independent samples per genotype. Data are presented as +/- SEM. ns = not significant, Student’s t-test.

For our study we used flies heterozygous for a previously characterized allele of ribosomal protein S13, *P[lacW]M (2)32A* (hereafter referred to as *rpS13/+* animals), which have decreased expression of *rpS13* mRNA (S1A Fig) and have been observed to have the classic *Minute* phenotype of shorter and thinner bristles and a delay in larval development [53]. We quantified the delay in development of *rpS13/+* and controls (*w*^*1118*^) by measuring the time it took for animals to reach the pupal stage after egg laying. We found that *rpS13/+* animals were delayed in development by about 40 hours, which corresponds to a delay of approximately 20% compared to control animals (Fig 1A). We also measured body size as the larvae developed, and we saw that *rpS13/+* larvae had a smaller size compared to age-matched control animals at different stages of larval development (S1B Fig). However, due to their prolonged larval period, the *rpS13/+* animals grew for a longer time. Hence, when we measured both final larval and pupal size, we found that, in both cases, *rpS13/+* animals were about 12% larger than controls (Figs 1B and 1C and S1C). We measured mouth hook movements as measure of feeding rate and saw a small, but significant, increase in *rpS13/+* larvae when compared to controls (S1D Fig). This indicates that the growth and developmental phenotypes of *rpS13/+* animals do not result simply from reduced feeding. These data suggest that *rpS13/+* animals exhibit a reduced growth and developmental rate.

Studies in different model systems have shown that the phenotypes seen in rp/+ animals are often associated with lowered ribosome numbers and reduced protein synthesis [21]. We therefore investigated ribosome levels and protein synthesis in *rpS13/+* animals. In order to measure ribosome numbers, we measured mature 18S and 28S rRNA in wandering L3 whole larval lysates. We saw no significant difference in rRNA levels between *rpS13/+* and control larvae (Fig 1D). Total protein content in wandering L3 larval lysates also showed no significant difference in *rpS13*/+ larvae compared to control larvae (Fig 1E). Finally, we investigated whether *rpS13/+* animals show a decrease in protein synthesis rate. To do this we used a puromycin labelling assay [54]. We first quantified the levels of puromycin incorporation of *rpS13/+* and control animals at the wandering larval stage in order to developmentally match control and *rpS13/+* animals and found no significant difference in protein synthesis rates (Fig 1F and 1G). We repeated this assay at two other earlier time points with aged-matched larvae, and once again found no decrease in translation rates in *rpS13*/+ larvae compared to control larvae (S2A and S2B Fig). This suggests that the *Minute* delayed development is not due to a global loss of ribosome numbers or translational capacity.

### *rpS13/+* animals show a defect in ecdysone signalling

**Fig 2:**
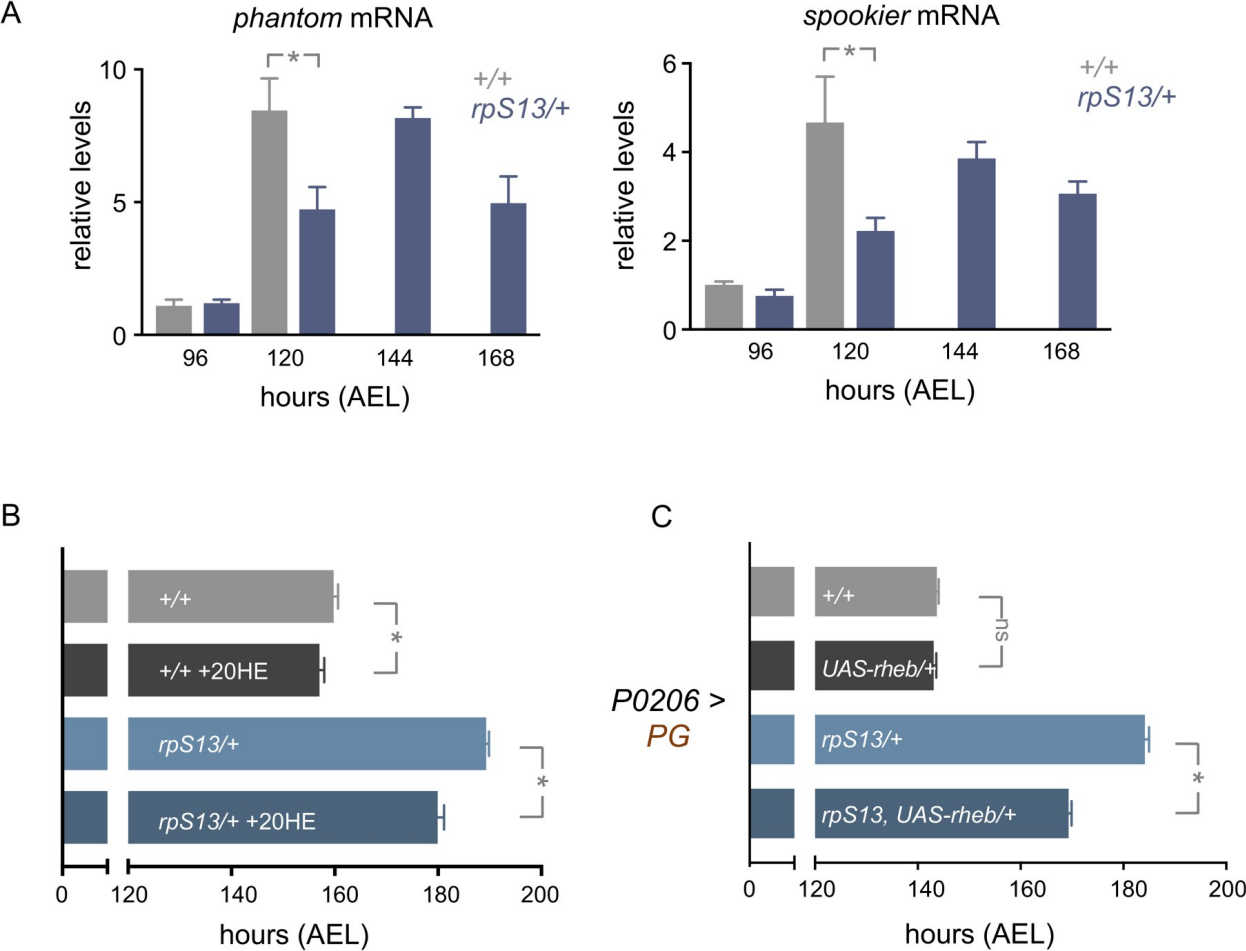
Delayed development in *rpS13/+* animals is due to impaired ecdysone production. (A) qRT-PCR measurements of *phantom* and *spookier* mRNA levels in *w*^*1118*^ and *rpS13/+* larvae. n = 4 independent samples per genotype. Data are presented as +/- SEM. *p < 0.05, two-way ANOVA and post-hoc Student’s t-test. (B) Time to pupation of *w*^*1118*^ and *rpS13/+* larvae grown in control food or food supplemented with ecdysone (20HE). Data are presented as +/- SEM. *p < 0.05, Mann-Whitney U test. n = 142 (*+/+*), 92 (*+/+* with 20HE), 148 (*rpS13/+*), 116 (*rpS13/+* with 20HE). (C) Time to pupation of *+/+* and *rpS13/+* larvae with or without *UAS-rheb* expression in the prothoracic gland using *P0206-Gal4*. Data are presented as +/- SEM. ns = not significant, *p < 0.05, Mann-Whitney U test. n = 152 (*+/*+), 157 (*UAS-rheb/+*), 151 (*rpS13/+*), 144 (*UAS-rheb/rpS13*).

The duration of the larval period is controlled in large part by the steroid hormone, ecdysone [27]. In particular, at the end of larval development, a neuro-endocrine circuit stimulates a pulse of ecdysone production and secretion from the prothoracic gland (PG). This circulating ecdysone then acts on larval tissues to trigger the larval to pupal transition. Any defects in this neuro-endocrine circuit leads to a delay in larval development to the pupal stage. Given their delayed development, we examined whether *rpS13/+* animals show a defect in ecdysone signaling. We did this by measuring the transcript levels of *phantom* and *spookier* both of which encode enzymes for PG ecdysone production. As previously described, both showed maximal expression peaks at 120 hours AEL in control animals, consistent with the ecdysone pulse that triggers pupation (Fig 2A). However, in *rpS13/+* animals these peaks were delayed by about one day (144 hours) and continued to show expression even at 168 hours for larvae that were still wandering (Fig 2A), suggesting a delay in ecdysone signalling. We also found that feeding larvae 20-hydroxyecdysone (20-HE) was able to partially reverse the development timing delay seen in the *rpS13/+* by about one third of the total delay (Fig 2B). Ecdysone synthesis in the PG can be stimulated by several different signaling pathways, including the Ras/ERK and TOR kinase pathways [32,55,56]. When we overexpressed the TOR activator, Rheb in the PG, we found that while it had no effect on developmental timing in control animals, it was sufficient to partially reverse the development timing delay seen in the *rpS13/+* animals again by about one third of the total delay (Fig 2C). Together, these data indicate that *rpS13/+* animals exhibit a delay in development that can be explained in part due to blunted ecdysone signaling.

### 5-HT neuronal *rpS13* is required for proper developmental timing

**Fig 3:**
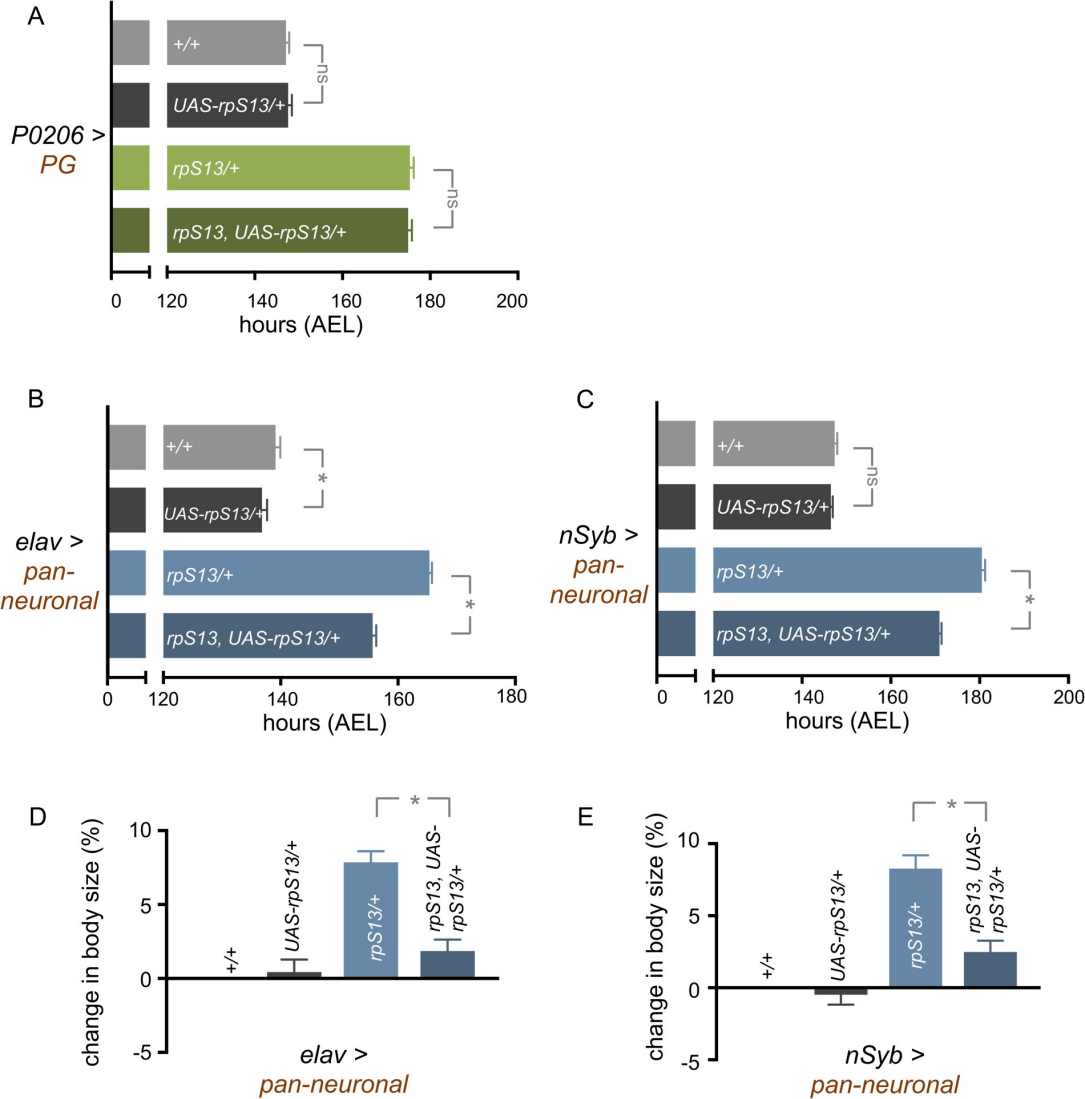
*rpS13* re-expression in neurons partially reverses the *rpS13/+* developmental delay. (A) Time to pupation of *+/+* and *rpS13/+* larvae with or without *UAS-rpS13* expression in the prothoracic gland using *P0206-Gal4*. Data are presented as +/- SEM. ns = not significant, Mann-Whitney U test. n = 95 (*+/*+), 95 (*UAS-rpS13/+*), 98 (*rpS13/+*), 87 (*rpS13*, *UAS-rpS13/+*). (B) Time to pupation of *+/+* and *rpS13/+* larvae with or without *UAS-rpS13* expression using the pan-neuronal driver, *elav-Gal4*. Data are presented as +/- SEM. ns = not significant, *p < 0.05, Mann-Whitney U test. *+/+* n = 180, *rpS13/+* n = 167, *UAS-rpS13/+* n = 182, *rpS13*, *UAS-rpS13/+* n = 226. (C) Time to pupation of *+/+* and *rpS13/+* larvae with or without *UAS-rpS13* expression using the pan-neuronal driver, *nSyb-Gal4*. Data are presented as +/- SEM. ns = not significant, *p < 0.05, Mann-Whitney U test. *+/+* n = 182, *rpS13/+* n = 180, *UAS-rpS13/+* n = 190, *rpS13*, *UAS-rpS13/+* n = 176. (D) Pupal volume of *+/+* and *rpS13/+* animals with or without *UAS-rpS13* expression using the pan-neuronal driver, *elav-Gal4*. Data are presented as +/- SEM. *p < 0.05, Student’s t-test. *+/+* n = 313, *rpS13/+* n = 468, *UAS-rpS13/+* n = 456, *rpS13*, *UAS-rpS13/+* n = 475. (E) Pupal volume of *+/+* and *rpS13/+* animals with or without *UAS-rpS13* expression using the pan-neuronal driver, *nSyb-Gal4*. Data are presented as +/- SEM. *p < 0.05, Student’s t-test. *+/+* n = 588, *rpS13/+* n = 484, *UAS-rpS13/+* n = 586, *rpS13*, *UAS-rpS13/+* n = 536.

We next examined whether the delayed development in *rpS13/+* animals reflects a more selective role for RpS13, perhaps in specific cells or tissues involved in controlling ecdysone. Our approach was to use the *Gal4/ UAS* system to re-express a RpS13 transgene *(UAS-rpS13)* in specific tissues in *rpS13/+* animals and then examine whether this could reverse the delay in development. We focused in particular on examining cells and tissues important for stimulating the late larval ecdysone pulse. We first re-expressed *UAS-rpS13* in the PG using the PG driver, *P0206-Gal4*. We saw no significant change in timing in control animals with the driver alone or with the over-expression of *UAS-rpS13* in control animals. Moreover, we found that PG-specific expression of *UAS-rpS13* in *rpS13/+* larvae was unable to reverse the delay in development seen in the *rpS13/+* animals (Fig 3A). We then examined the imaginal discs. Reducing Rp expression in imaginal discs can elevate dilp8 levels [49,51], and a previous study showed that the developmental delay seen in *rpS3/+ Minute* larvae can be partially reversed in a *dilp8* heterozygous background [51]. We found that *rpS13/+* larvae also had increased imaginal disc expression of a dilp8-GFP reporter particularly in leg discs and the wing disc pouch (S3A Fig), but the delayed development in *rpS13/+* larvae was unaffected in a *dilp8* heterozygous mutant background (S3B Fig). We also examined the effects of imaginal disc RpS13 expression on developmental timing in *rpS13/+* larvae. We used the *esg-Gal4*^*ts*^ which directs temperature-inducible expression in all imaginal cells, including the discs, in the larvae. We found that expression of *UAS-rpS13* throughout larval development using *esg-Gal4*^*ts*^ had little effect on developmental timing in control animals, but instead had an exacerbation of developmental delay in *rpS13/+* animals (S3C Fig). A similar, although weaker effect, was seen with the imaginal disc driver *nubbin-Gal4* (S3D Fig).

The PG-induced expression of ecdysone at the end of the larval period is controlled by neuronal signals to the PG. We therefore examined the effect of re-expressing *rpS13* in neurons using a pan-neuronal driver, *elav-Gal4*. We found that while neuronal expression of *UAS-rpS13* in the control animals did not affect timing, it was sufficient to partially rescue the developmental delay in *rpS13/+* larvae (Fig 3B). Indeed, this rescue (~30–40%) was similar to that seen with either 20HE feeding or by stimulation of TOR signaling in the PG (see Fig 2B and 2C). We confirmed this result by using a second pan-neuronal driver, *nSyb-Gal4*, which also rescued timing by roughly one third while not accelerating timing in the wild type animals (Fig 3C). Since *rpS13/+* animals also have an increased final body size phenotype, we measured pupal volume in animals with neuronal *UAS-rpS13* expression. We found that expression of *UAS-rpS13* in the *rpS13/+* larvae with either *elav-Gal4* or *nSyb-Gal4* led to a significant reversal of the increased body size seen in *rpS13/+* animals (Fig 3D and 3E). These data indicate that a neuronal requirement for RpS13 in larval development can, in part, account for the delayed development seen in *rpS13/+* animals. These effects are likely not due to alterations in brain size because we found no difference in wandering larval brain size (ventral nerve cord width) between *rpS13/+* and control animals (S4 Fig)

**Fig 4:**
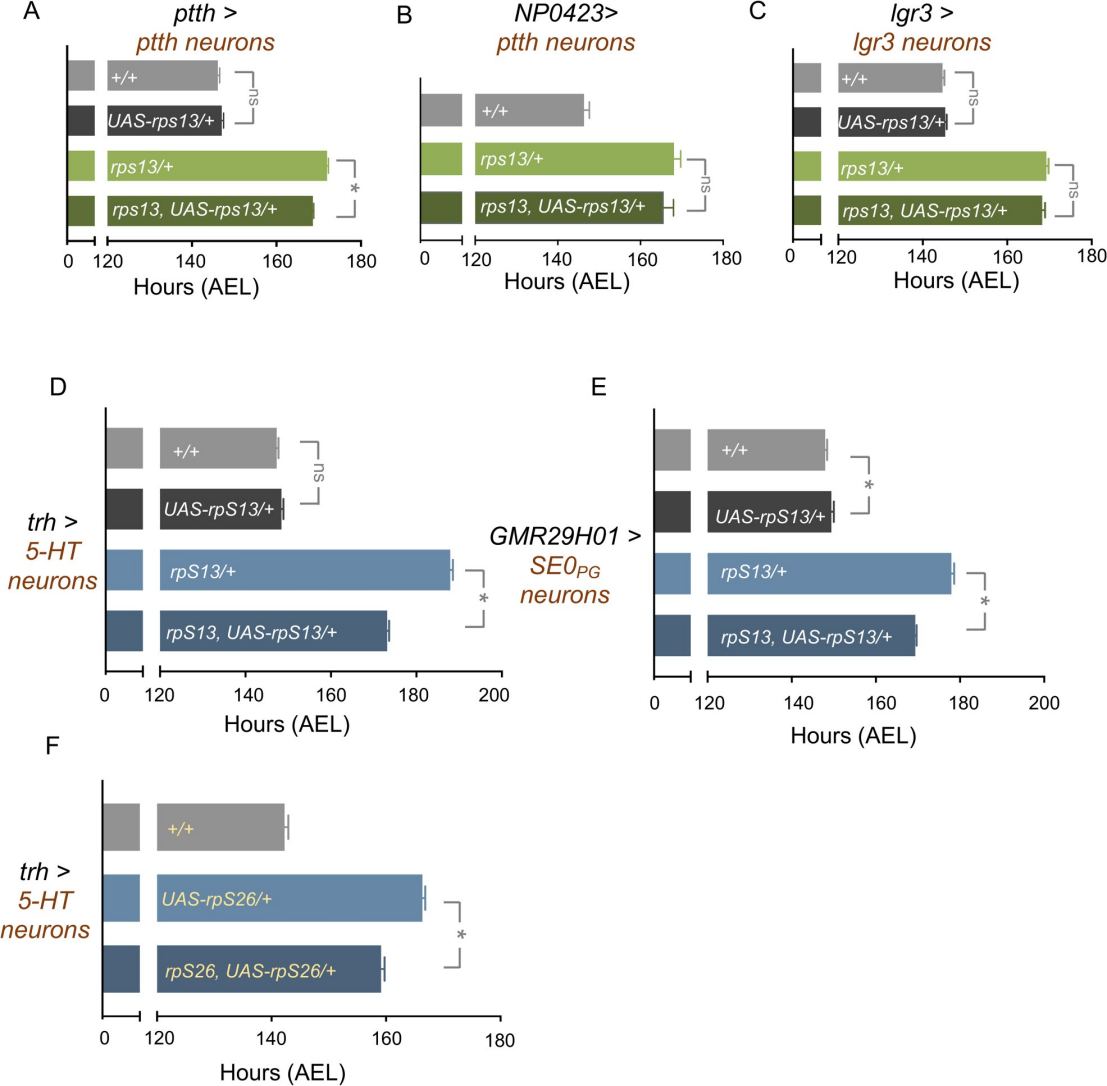
*rpS13* re-expression in the serotonergic neurons that innervate the prothoracic gland partially rescues *rpS13/+* developmental delay. (A) Time to pupation of *+/+* and *rpS13/+* larvae with or without *UAS-rpS13* expression in the ptth neurons using *ptth-Gal4*. Data are presented as +/- SEM. ns = not significant, *p < 0.05, Mann-Whitney U test. n = 233 (*+/*+), 288 (*UAS-rpS13/+*), 282 (*rpS13/+*), 282 (*rpS13*, *UAS-rpS13/+*). (B) Time to pupation of *+/+* and *rpS13/+* larvae with or without *UAS-rpS13* expression in the ptth neurons using *NP0423-Gal4*. Data are presented as +/- SEM. ns = not significant, *p < 0.05, Mann-Whitney U test. n = 127 (*+/*+), 81 (*rpS13/+*), 84 (*rpS13*, *UAS-rpS13/+*). (C) Time to pupation of *+/+* and *rpS13/+* larvae with or without *UAS-rpS13* expression in Lgr3-expressing neurons using *lgr3-Gal*. Data are presented as +/- SEM. ns = not significant, *p < 0.05, Mann-Whitney U test. n = 92 (*+/*+), 176 (*UAS-rpS13/+*), 177 (*rpS13/+*), 166 (*rpS13*, *UAS-rpS13/+*). (D) Time to pupation of *+/+* and *rpS13/+* larvae with or without *UAS-rpS13* expression in serotonergic neurons using *Trh-Gal4*. Data are presented as +/- SEM. ns = not significant, *p < 0.05, Mann-Whitney U test. n = 241 (*+/*+), 176 (*UAS-rpS13/+*), 234 (*rpS13/+*), 230 (*rpS13*, *UAS-rpS13/+*). (E) Time to pupation of *+/+* and *rpS13/+* larvae with or without *UAS-rpS13* expression in SE0_PG_ serotonergic neurons using *GMR29H01-Gal4*. Data are presented as +/- SEM. *p < 0.05, Mann-Whitney U test. n = 225 (*+/*+), 196 (*UAS-rpS13/+*), 192 (*rpS13/+*), 248 (*rpS13*, *UAS-rpS13/+*). (F) Time to pupation of *+/+* larvae and *rpS26/+* larvae with or without *UAS-rpS26* expression in serotonergic neurons using *Trh-Gal4*. Data are presented as +/- SEM. *p < 0.05, Mann-Whitney U test. n = 116 (*+/*+), 111 (*rpS26/+*), 184 (*rpS26*, *UAS-rps26/+*).

We next examined whether the requirement for neuronal RpS13 for proper developmental timing might reflect a role in a specific subset of neurons, particularly those known to influence PG function. One important subset is a pair of bilateral PTTH-expressing neurons that directly innervate the PG. These respond to developmental cues to secrete the peptide PTTH which acts on the PG to stimulate peak levels of ecdysone biosynthesis at the end of the larval stage [29,30]. We therefore examined the effects of expression of *UAS-rpS13* in the PTTH neurons using two drivers that express in these cells, *ptth-Gal4* and *NP0423-Gal4*. We found that when we expressed *UAS-rpS13* with *ptth-Gal4* we saw no effect on developmental timing in control animals and a small rescue of the developmental delay in *rpS13/+* animals (Fig 4A). In contrast, expression of *UAS-rpS13* with *NP0423-Gal4* had no significant effect on the developmental delay in *rpS13/+* animals (Fig 4B). The PTTH neurons are also themselves directly regulated by another subset of neurons (Lgr3-expressing neurons) that are controlled by tissue damage-induced secretion of dilp8 [33–35]. Expression of *UAS-rpS13* using the *lgr3-Gal4* driver had no effect on developmental timing either in control or *rpS13/+* animals (Fig 4C). These results suggest that expression of the RpS13 in neurons that control PTTH signaling does not fully account for the rescue of *Minute* developmental timing that we observed with pan neuronal RpS13 expression.

The PG is also directly innervated by serotonergic (5-HT) neurons. These 5-HT neurons are required for proper ecdysone production at the end of the larval stage, particularly in response to dietary nutrients [31]. When we used the 5-HT neuronal driver *trh-Gal4* to express *UAS-rpS13* we found that we could reverse the delay in development seen in *rpS13/+* animals by about one-third (Fig 4D). This recapitulates the extent of the rescue seen with pan-neuronal *UAS-rpS13* expression (see Fig 3B and 3C) and is similar to that seen with either 20HE feeding or by TOR-dependent activation in the PG (see Fig 2B and 2C). There are approximately 100 serotoninergic neurons in the larval brain, of which three pairs—termed SE0_PG_ neurons—innervate the prothoracic gland directly [31]. Using another neuronal driver which has a more limited expression pattern which includes the SE0_PG_ neurons (*GMR29H01-Gal4)* we re-expressed *UAS-rpS13* in *rpS13/+* and control animals and found that timing was again rescued by approximately one-third in the *rpS13/+* animals while development was unaffected in control animals (Fig 4E). We also examined a second *Minute* line, *rpS26*^*04553*^, and found expression of a *UAS-rpS26* transgene using *trh-GAL4* could also partially reverse the delayed larval development in *rpS26/+* animals (Fig 4F). These data suggest the *Minute* phenotype, in part, reflects a specific role for Rp function in 5-HT neurons that innervate the PG in the regulation of developmental timing.

### Serotonergic alterations in protein synthesis or proteostasis do not alter developmental timing

**Fig 5:**
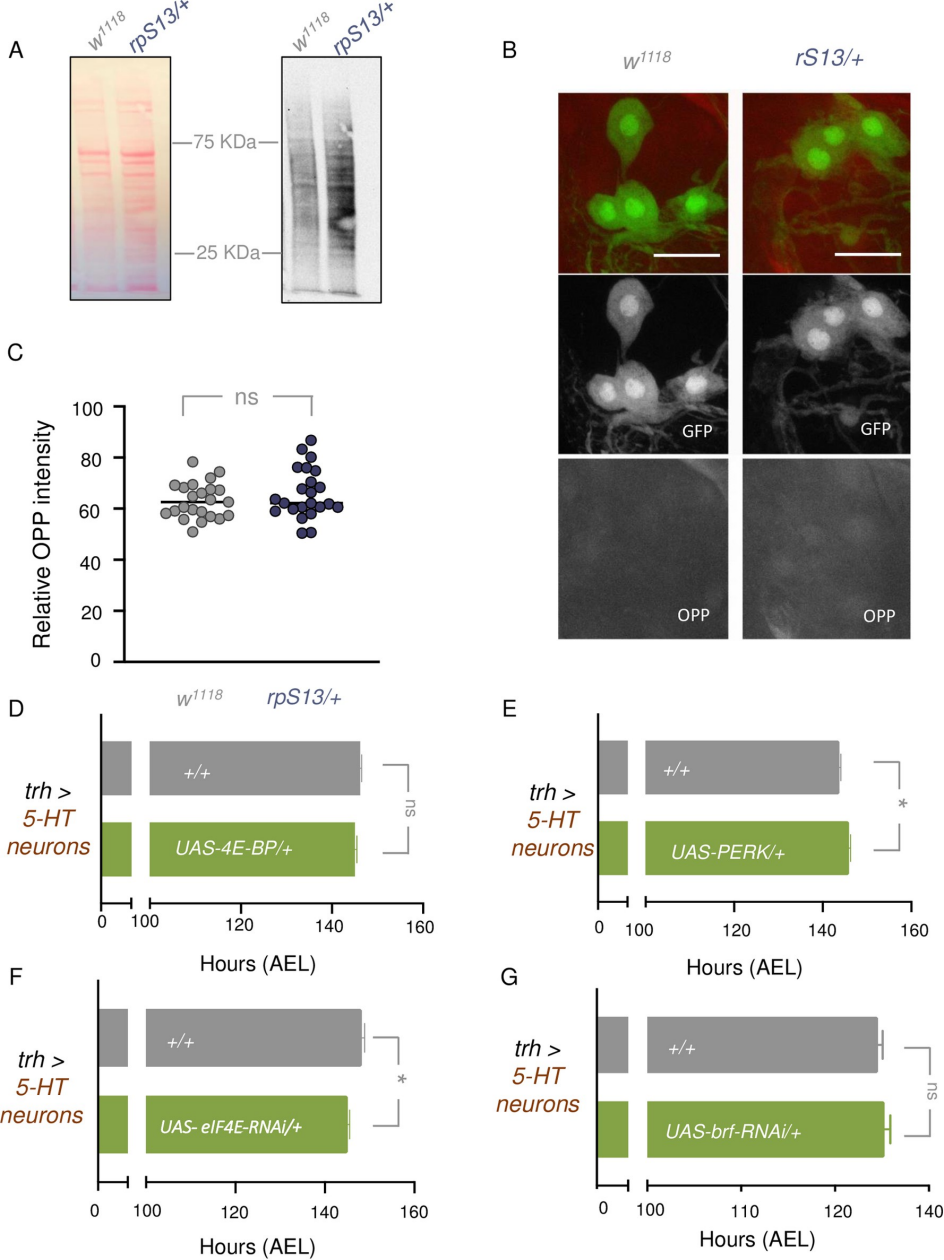
Inhibiting translation in 5-HT neurons does not recapitulate a *Minute* phenotype. (A) Puromycin labelling of *w*^*1118*^ and *rpsS13/+* third instar wandering larvae brains. Left, Ponceau S staining showing total protein. Right, anti-puromycin immunoblot. (B) OPP incorporation in 5-HT neurons, marked by GFP, from wandering L3 larvae. Scale bars, 10μm. (C) Quantification of OPP intensity in GFP marked 5-HT cells. *+/+* n = 23, *rpS13/+* n = 23. Individual data points are plotted, and the bars represent mean +/- SEM. ns = not significant, Student’s t-test. (D) Time to pupation of control larvae and larvae with *UAS- 4E-BP* overexpression in 5-HT neurons using *trh-gal4*. *+/+* n = 212, *+/ UAS-4E-BP* n = 141. Data are presented as +/- SEM. ns = not significant, Mann-Whitney U test. (E) Time to pupation of control larvae and larvae with *UAS- PERK* overexpression in 5-HT neurons using *trh-gal4*. *+/+* n = 242, *+/ UAS-PERK* n = 256. Data are presented as +/- SEM. *p < 0.05, Mann-Whitney U test. (F) Time to pupation of control larvae and larvae with RNAi knockdown of *eIF4E* in 5-HT neurons using *trh-gal4*. *+/+* n = 208, *+/ UAS-eIF4E-RNAi*. n = 196. Data are presented as +/- SEM. *p > 0.05, Mann-Whitney U test. (G) Time to pupation of control larvae and larvae with RNAi knockdown of *brf* in 5-HT neurons using *trh-gal4*. *+/+* n = 110, *+/ UAS-brf-RNAi*. n = 116. Data are presented as +/- SEM. ns = not significant, Mann-Whitney U test.

Although we saw no changes in whole-body protein synthesis in our *rp/+* animals (Fig 1), we explored the possibility that selective reductions in neuronal protein synthesis might explain the *Minute* developmental delay. When we measured wandering larval brain translation rates using puromycin labelling we saw no decrease in the *Minute* animals (Fig 5A). We also examined protein synthesis specifically in the SE0_PG_ neurons using O- propargyl-puromycin (OPP) incorporation. In general, we saw uniformly low OPP incorporation in cells across the brain compared to other larval tissues such as the prothoracic glad (S5 Fig). However, we saw no difference in OPP incorporation in the GFP-marked SE0_PG_ neurons in controls vs. *rpS13/+* animals (Fig 5B and 5C). Thus, we do not find any evidence of reduced protein synthesis in 5-HT neurons of *rpS13/+* animals compared to controls.

We next examined the effects of genetic manipulation of protein synthesis in 5-HT neurons. We reasoned that if the developmental delay in *rp/+* larvae is due to reduced protein synthesis we might be able to recapitulate a *Minute* phenotype in wild-type animals by genetically inhibiting translation in the 5-HT neurons. We began by overexpressing two known repressors of translation - 4E-BP, which binds to and inhibits the translation initiation factor eIF4E, and PERK, a kinase that phosphorylates the translational initiation factor eIF2 alpha. We overexpressed both 4EBP and PERK in 5-HT neurons using *trh-gal4* and examined effects on developmental timing. We found that overexpression of 4EBP had no effect, while PERK overexpression induced a minor delay in development (Fig 5D and 5E). We also examined the effects of RNAi-mediated knockdown of eIF4E and the Pol III transcriptional factor Brf, which is required for tRNA synthesis, using *trh-gal4*. In both cases, we saw no delay in larval development (Fig 5F and 5G). These results together suggest that the *Minute* phenotype of *rpS13/+* animals are not due to alterations in bulk translation in the 5-HT neurons.

**Fig 6:**
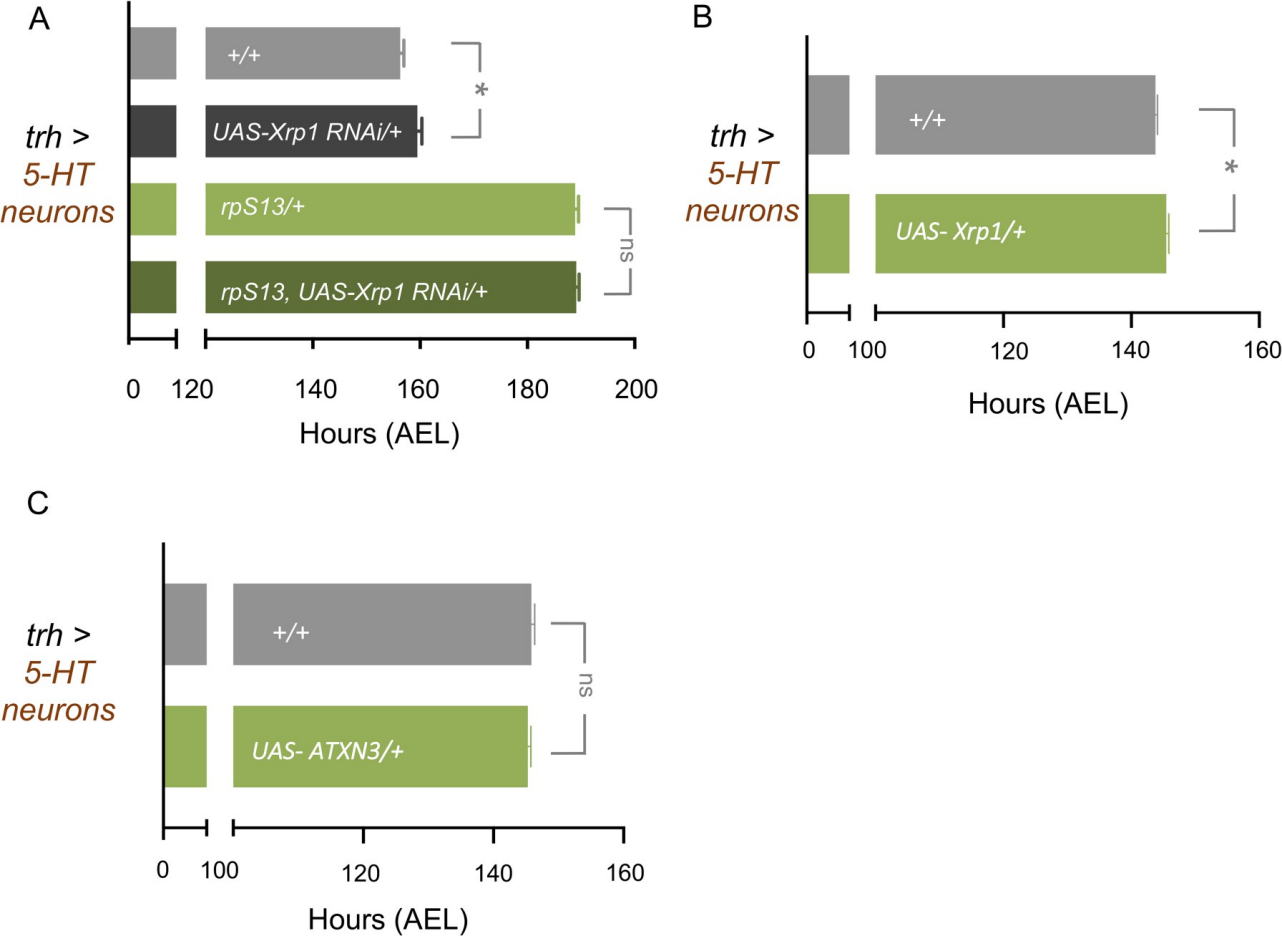
Inducing proteotoxic stress in 5-HT neurons does not recapitulate a *Minute* phenotype. (A) Time to pupation of *+/+* and *rpS13/+* larvae with or without RNAi knockdown of *Xrp1 (UAS-Xrp1 RNAi)* in serotonergic neurons using *Trh-Gal4*. *+/+* n = 200, *UAS-Xrp1RNAi/+* n = 160, *rpS13/+* n = 179, *UAS-Xrp1RNAi/rpS13* n = 203. Data are presented as +/- SEM. *p < 0.05, Mann-Whitney U test. (B) Time to pupation of control larvae and larvae with *UAS- Xrp1* overexpression in 5-HT neurons using *trh-gal4*. *+/+* n = 242, *+/ UAS-Xrp1* n = 202. Data are presented as +/- SEM. *p < 0.05, Mann-Whitney U test. (C) Time to pupation of control larvae and larvae with *UAS- ATXN3* overexpression in 5-HT neurons using *trh-gal4*. *+/+* n = 162, *+/ UAS-ATXN3* n = 163. Data are presented as +/- SEM. ns = not significant, Mann-Whitney U test.

Recent studies on cell competition have described how elimination of *rp/+* cells is primarily triggered by proteotoxic stress rather than reduced translation rates[41,42]. One mechanism involves induction of the transcription factor *Xrp1* which has been shown to be an effector of *Minute* cell competition in imaginal discs through its ability to both sense and induce proteotoxic stress and to reduce protein synthesis [50,57,58]. We therefore examined a role for Xrp1 and proteostasis in the 5-HTergic control of development in *rp/+* animals. We first used RNAi to knockdown *Xrp1* specifically in 5-HT neurons to test whether this could reverse the delayed development in *rpS13/+* animals. However, we found that the extended developmental timing in the *rpS13/+* animals was unaffected by *Xrp1* knockdown (Fig 6A). We also examined the effects of overexpression of *UAS-Xrp1* in 5-HT neurons using *trh-gal4*. We saw that Xrp1 overexpression induced a small delay in development and did not mimic the strong developmental delay seen in *Minute* larvae (Fig 6B). Finally, we tested the effects of ectopic expression of ataxin-3, a human aggregate-prone polyQ protein in 5-HT neurons. A previous report showed that overexpression of this protein in wing disc cells could mimic both the proteostasis and cell competitive elimination seen in rp/+ cells [42]. However, we did not find any difference in developmental timing between control animals and those with ATXN3 expressed in 5-HT neurons (Fig 6C).

### The developmental delay in *rpS13/+* animals is partially reversed by serotonergic expression of synaptic vesicle proteins

**Fig 7:**
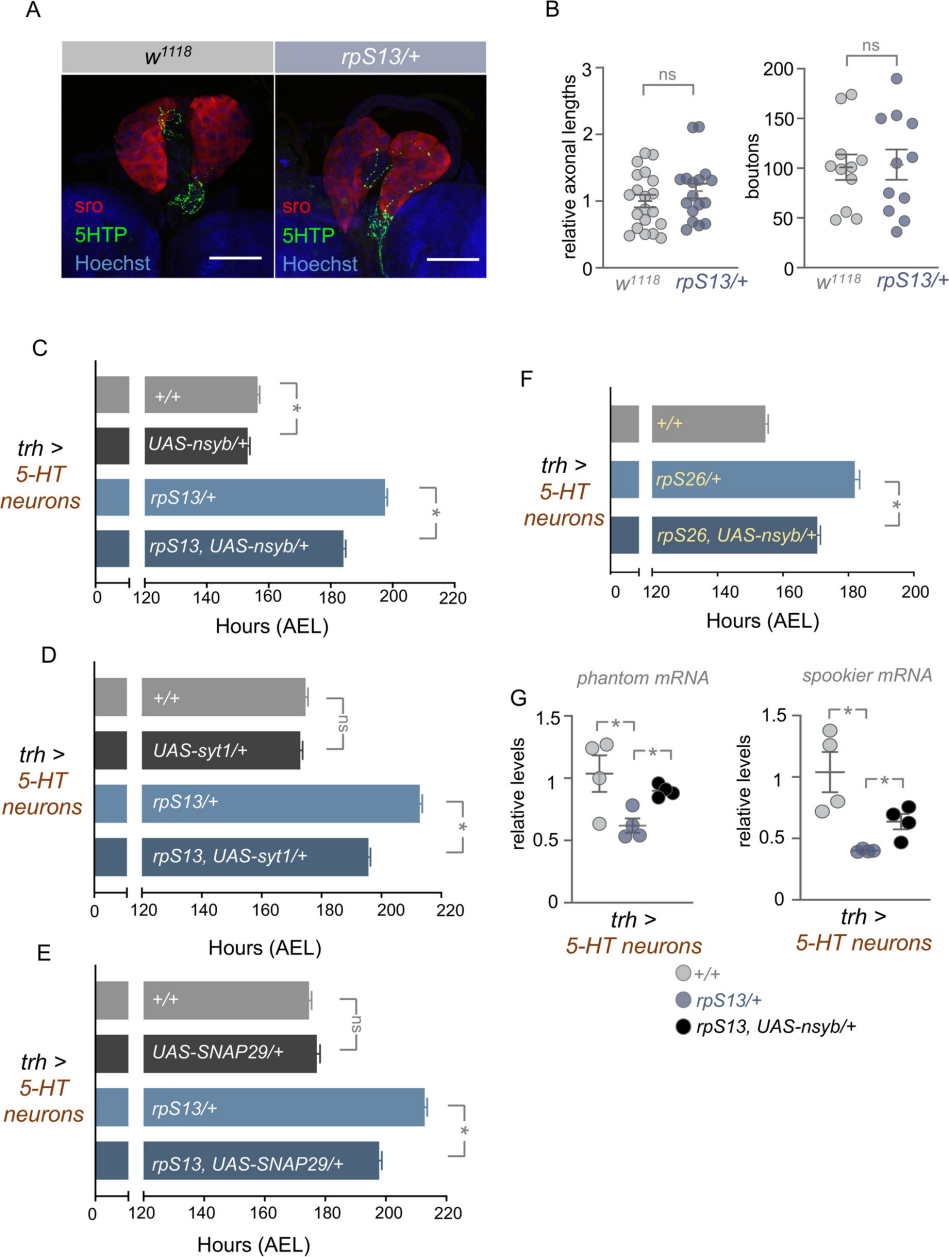
Expression of individual SNARE complex proteins partially rescues *rpS13/+* developmental delay. (A) Fluorescent confocal images of representative *+/+* and *rpS13/+* prothoracic glands showing innervation of serotonergic neurons. Anti- 5-HTP is labelled with GFP, while the prothoracic gland is labelled in red using an anti-shroud antibody. Nuclei of both the brain and prothoracic gland are stained with Hoechst. Scale bars, 50 μm. (B) Quantification of relative axon lengths (left) and bouton numbers (right) of 5-HT neurons that overlap the prothoracic gland. Individual data points are plotted, and the bars represent mean +/- SEM. ns = not significant, Student’s t-test. (C) Time to pupation of *+/+* and *rpS13/+* larvae with or without *UAS-nsyb* overexpression in serotonergic neurons using *Trh-Gal4*. *+/+* n = 182, *UAS-nsyb/+* n = 180, *rpS13/+* n = 190, *rpS13*, *UAS-nsyb/+* n = 176. Data are presented as +/- SEM. *p < 0.05, Mann-Whitney U test. (D) Time to pupation of *+/+* and *rpS13/+* larvae with or without *UAS-syt1* overexpression in serotonergic neurons using *Trh-Gal4*. *+/+* n = 168, *UAS-syt1/+* n = 151, *rpS13/+* n = 137, *rpS13*, *UAS-syt1/+* n = 136. Data are presented as +/- SEM. ns = not significant, *p < 0.05, Mann-Whitney U test. (E) Time to pupation of *+/+* and *rpS13/+* larvae with or without *UAS-SNAP29* overexpression in serotonergic neurons using *Trh-Gal4*. *+/+* n = 168, *UAS-SNAP29/+* n = 140, *rpS13/+* n = 137, *rpS13*, *UAS-SNAP29/rpS13* n = 135. Data are presented as +/- SEM. ns = not significant, *p < 0.05, Mann-Whitney U test. (F) Time to pupation of *+/+* larvae and *rpS26/+* larvae with or without *UAS-nsyb* overexpression in serotonergic neurons using *Trh-Gal4*. *+/+* n = 182, *rpS26/+* n = 190, *rp26*, *UAS-nsyb/+* n = 176. Data are presented as +/- SEM. *p < 0.05, Mann-Whitney U test. (G) qRT-PCR measurements of *phantom* and *spookier* mRNA levels in *+/+* larvae and *rpS13/+* larvae with or without *UAS-nsyb* overexpression in serotonergic neurons using *Trh-Gal4*. Individual data points are plotted, and the bars represent mean +/- SEM. p > 0.05, One-way ANOVA and post-hoc Student’s t-test.

We next examined whether specific aspects of the biology of 5-HT neurons are impaired in *rp/+* animals. A previous report showed that the 5-HT neurons that project to the PG are regulated by nutrient availability and that in low nutrient conditions these neuronal projections are reduced, leading to diminished 5-HT signaling to the PG and, as a result, reduced ecdysone release and delayed development [31]. We therefore investigated whether *rpS13/+* animals also showed a reduction in axonal projections into the PG. We stained 5-HT neurons in both control and *rpS13/+* animals and in both cases, we observed projections to the PG. When we measured relative axon lengths or bouton numbers of 5-HT neurons that cover the PG we found no difference between controls and *rpS13/+* animals, suggesting no alterations in 5-HT neuronal outgrowth (Fig 7A and 7B). It is possible that the 5-HT neurons in *rpS13/+* larvae have reduced activity, leading to decreased stimulation of ecdysone production in the PG. To explore this possibility, we examined the effects of genetic activation of these neurons by expressing the bacterial sodium channel (NaChBac), which leads to depolarization and activation of neurons. However, we found that expression of *UAS-NaChBac* did not reverse the delay in development seen in *rpS13/+* animals (S6A Fig). We also found that expression of tryptophan hydroxylase (Trh), a key enzyme in the synthesis of 5-HT, also did not reverse the delayed development in *rpS13/+* larvae (S6B Fig).

**Fig 8:**
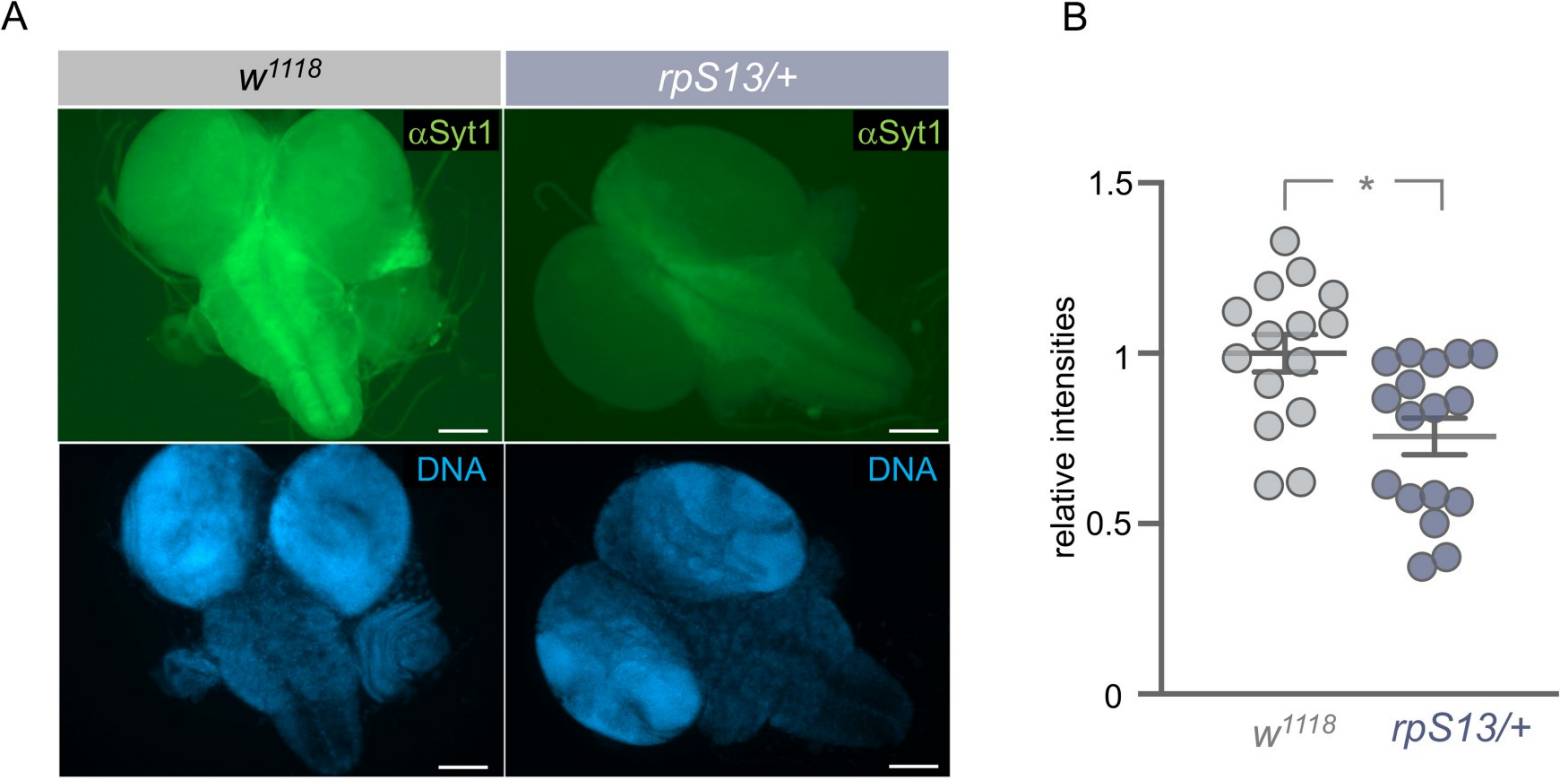
*rpS13/+* have reduced brain synaptotagmin (Syt1) levels. (A) Brains of *w*^*1118*^ and *rpS13/+* wandering third-instar larvae stained with anti-Syt1 antibody (green) and Hoechst 33258 (blue). Scale bars, 100 μm (B) Quantification of relative intensities of anti-Syt1 antibody staining in *w*^*1118*^ and *rpS13/+* brains. Individual data points are plotted, and the bars represent mean +/- SEM. *p > 0.05, Student’s t-test.

A key process in neurons is the vesicle mediated secretion of neurotransmitters and neuropeptides. The synthesis, axonal transport, and synaptic release of vesicle contents is controlled by several proteins, including members of the SNAP Receptor (SNARE) complex. Interestingly, previous studies have shown that these synaptic vesicle proteins need to be continually synthesized for proper neuronal function [59] and that their synthesis is often translationally regulated [60,61]. We therefore examined the effects of Gal4-mediated overexpression of three synaptic vesicle proteins–*UAS-nsyb*, *UAS-syt1*, *UAS-SNAP29—*in serotonergic neurons. Strikingly, we found that individual expression of all three synaptic vesicle proteins was able to rescue developmental delay seen in *rpS13/+* animals, to the same magnitude as that seen with expression of *UAS-S13* (Fig 7C–7E). Furthermore, we saw that serotonergic expression of *UAS-nsyb* was also able to partially reverse the developmental delay seen in both *rpS26/+* and *rpS24/+ Minute* larvae (Figs 7F and S7). Finally, we found that the reduced expression of the ecdysone synthesis genes, *phantom* and *spookier* that was observed in *rpS13/+* larvae compared to control larvae was partially reversed by serotonergic neuron overexpression of *UAS-nsyb* (Fig 7G). These data suggest that a potential mechanism by which rpS13 in 5-HT neurons controls the *Minute* phenotype is through regulation of synaptic vesicle production or function, which impacts the regulation of PG-synthesis of ecdysone. To test this further, we examined whether *Minute* animals might show altered expression of synaptic vesicle proteins. We used immunostaining to Syt1—one of the SNARE complex proteins whose overexpression can partially reverse the *Minute* developmental delay—in control and *rpS13/+* larval brains. In the control animals we saw strong Syt1 staining in the neuropil of the brain, as has previously been seen with synaptic vesicle proteins [62]. However, this expression was significantly lower in the *rpS13/+* brains (Fig 8A and 8B).

## Discussion

Since the discovery that *Minutes* are mutants for Rps, a prevailing hypothesis (the “balance hypothesis”) has been that their developmental phenotypes result from an imbalance of cytoplasmic ribosomal protein concentrations leading to incomplete ribosomal subunit assembly and reduced overall translation [39]. However, our data indicate that despite all cells in a *Minute* animal being heterozygous for an Rp gene, this does not result in a whole-body decrease in ribosome numbers or protein synthesis. Rather, the developmental delay phenotypes in *Minute* animals likely result from more selective effects of Rp loss. Here we identify 5-HT neurons as one important cell-type in which Rp function is required for proper larval developmental timing.

Interestingly we found that the effect of RpS13 in 5-HT neurons could account for 30–40% of the total developmental delay in *rpS13/+* animals. Part of the delay may be attributed to the overall slower growth (mass increase) in *rp/+* animals that results in them taking longer to reach critical weight to allow for subsequent pupariation. In addition, it is also possible that RpS13 containing ribosomes may be playing roles in other tissues to specifically control post-critical weight larval maturation. Indeed, previous studies have also shown tissue-selective effects of Rps on development. For example, *rpS3/+* larvae have elevated imaginal disc expression of dilp8, which can signal to the brain to suppress PG production of ecdysone and delay development. Indeed, loss of one copy of *dilp8*, was shown to be sufficient to partially reverse the delayed development in *rpS6/+* larvae [51]. A previous study also found that re-expression of *rpS6* in prothoracic glands could partially rescue the animal’s developmental delay in *rpS6/+* larvae [52]. One possibility is that Rp function in a combination of tissues is required for proper endocrine control of developmental timing. Further studies are required to see if these tissue-specific contributions are similar for all Rps or may show some heterogeneity depending on the Rp being examined. For example, we found that expression of RpS13 in the prothoracic gland had no effect on the delayed development in *rpS13/+* larvae and actually further enhanced this delay when expressed in imaginal discs, possibly due to even higher induction of dilp8, suggesting that the 5-HT phenotype that we discovered may be particularly important for the *rpS13 Minute* developmental delay phenotype. The 5-HT neurons that innervate the PG have been shown to be particularly important in coupling nutrition to the control of ecdysone and developmental timing [31]. Given that the control of ribosome synthesis is nutrient-regulated [63], our results pinpointing a key role for Rps in the function of these neurons suggest one way that nutrients may modulate the 5-HT control of ecdysone.

We found that the developmental delay seen in *rp/+* larvae occurred independently of two processes proposed to underlie *rp/+* cell competition–impaired translation and induction of proteostasis. Instead, we found the delay in development could be partially reversed by overexpression of synaptic vesicle proteins. This finding raises the possibility that the requirement for RpS13 in 5-HT neurons may reflect a role in the production or function of synaptic vesicles. Current models suggest that rp/+ phenotypes arise from either heterogeneous ribosomes or selective mRNA translational control [19–23]. Therefore, one possibility is that the delayed development of *rpS13/+* is due to a selective change in mRNA translation of proteins involved in controlling neuronal secretory function. Indeed, we saw that the protein level of one synaptic vesicle protein, Syt1, was reduced in *rpS13/+* brains. In mammals, synaptic vesicle proteins are required to be continually synthesized for proper neuronal function [59]. Moreover, their expression has been shown to be post-transcriptionally regulated [60]. Hence, the vesicle proteins themselves may be subject to selective translational regulation. It could be that SNARE complex mRNAs share a common feature in their 5’ or 3’ UTR regions that leads to them being translationally regulated in similar ways. Indeed, UTR-mediated regulation of translation is a prevalent mode of controlling gene expression in neurons [64]. The subcellular control of translation is also important in neurons [65–67]. Here, local translation in regions such as cell bodies, axons and termini allows for selective, spatial control over protein synthesis. For example, it has been found that SNAP25 synaptic vesicle proteins can be locally translated in axons [61]. Thus, it is possible that the defects in *Minute* animals may occur due to alterations in local translation in 5-HT neurons.

Our findings may also be relevant to human biology. It has been observed that many neurological disorders arise from abnormal synaptic vesicle formation and function. These “synaptopathies” include disorders such as schizophrenia, AHDH, and autism, and are often associated with aberrant mRNA translation [68,69]. Interestingly some ribosomopathies in humans have also been shown to present with various neurological disorders such as microcephaly and mental retardation. In particular, mutations in *rpS23* and *rpL10* have been shown to be associated with autism spectrum disorder [70,71], a disorder that has been associated with aberrant 5-HT function [72]. Based on our findings, we can speculate that certain ribosomopathies that present with neural disorders may in part be due to defects in 5-HT neuronal vesicle function and, as a result, disrupted synaptic function.

## Materials and methods

### Fly strains and husbandry

Flies were raised on food with the following composition: 150 g agar, 1600 g cornmeal, 770 g torula yeast, 675 g sucrose, 2340 g D-glucose, 240 ml acid mixture (propionic acid/phosphoric acid per 34 liters of water). For all experiments larvae were maintained at 25°C, unless otherwise indicated. Unless otherwise stated the fly strains used were obtained from the Bloomington Stock Center (BDSC): *w*^*1118*^, yw, yw*;P[w[+mC] = lacW]rpS13[1]/CyO (BDSC 2246), *rpS26*^*04553*^*/CyO* (BDSC 12048), *rpS24*^*f06717*^*/CyO* (BDSC 19002), *UAS-rpS13GFP/cyo* (Gift from S. Brogna), *UAS-RpS26 (*FlyORF F000865), *P0206-Gal4* (Gift from C. Mirth), *esg*^*ts*^*-Gal4*, *elav-Gal4* (Gift from F.Buldoc), *nsyb-Gal4* (BDSC 51635), *ptth-Gal4* (Gift from M. O’Connor), *nubbin-Gal4* (BDSC 86108), *dilp8-GFP* (*dilp8*^*MI00727*^ eGFP trap BDSC 33079), *trh-Gal4* (BDSC 38388), *GMR29H01-Gal4* (BDSC 47343), *UAS-NaChBac* (BDSC 9469), *UAS-Rheb (*BDSC 9688), *UAS-trh* (BDSC 27638), *UAS-nsybGFP* (BDSC 6921, 6922), *UAS-syt1* (BDSC 6925), *UAS-SNAP29* (BDSC 56817), *P0206-Gal4* (Gift from M. O’Connor), *lgr3-Gal4* (BDSC 66683), *UAS-Xrp1RNAi* (BDSC 34521), *yv*: *attp2* (BDSC 36303), *UAS-4EBP* (BDSC 77999), *UAS-PERK* (BDSC 7608), *UAS-brf-RNAi* (BDSC 29321), *UAS-eIF4E-RNAi* (Vienna Drosophila Research Centre 7800), *UAS-ATXN3* (BDSC 8149), *UAS-Xrp1* [73], *act>CD2-gal4; UAS-GFP* (Gift from B. Edgar). All *Minute* alleles were backcrossed into our lab *w*^*1118*^ stock for six generation, and this *w*^*1118*^ was used as a control line. For all UAS experiments, the control cross used the relevant background for the UAS transgenic line (either *w*^*1118*^, *yw* or *TRiP* control).

### Measurement of *Drosophila* development and body size

For measuring developmental timing to pupal stage, newly hatched larvae were collected at 24 hr AEL and placed in food vials (50 larvae per vial) and kept at 25°C. The number of pupae was counted at least twice a day. For each experimental condition, a minimum of four replicates was used to calculate the mean time to develop into pupae. To measure pupal volume, pupae were imaged using a Zeiss Discovery V8 Stereomicroscope with Axiovision imaging software. Pupal length and width were measured, and pupal volume was calculated using the formula, volume = 4/3π(L/2) (l/2)2. A minimum of four replicates was used to calculate the mean volume for each genotype.

### Quantitative PCR

Total RNA was extracted from larvae (groups of 10) using TRIzol according to manufacturer’s instructions (Invitrogen; 15596–018). RNA samples were then subjected to DNase treatment according to manufacturer’s instructions (Ambion; 2238 G) and reverse transcribed using Superscript II (Invitrogen; 100004925). The generated cDNA was used as a template to perform qRT–PCRs (ABI 7500 real time PCR system using SyBr Green PCR mix) using specific primer pairs. PCR data were normalized to either actin or alpha-tubulin levels. The following primers were used:

RpS13 forward: AGGCAGTGCTCGACTCGTAT
RpS13 reverse: TTCCCGAGGATCTGTACCAC

Beta-tubulin forward: ATCATCACACACGGACAGG
Beta-tubulin reverse: GAGCTGGATGATGGGGAGTA

Actin5C forward: GAGCGCGGTTACTCTTTCAC
Actin5C reverse: GCCATCTCCTGCTCAAAGTC

18S rRNA forward: CCTGCGGCTTAATTTGACTC
18S rRNA reverse: ATGCACCACCACCCATAGAT

28S rRNA forward: TGCCAGGTAGGGAGTTTGAC
28S rRNA reverse: CAAGTCAGCATTTGCCCTTT

spookier forward: TATCTCTTGGGCACACTCGCTG
spookier reverse: GCCGAGCTAAATTTCTCCGCTT

phantom forward: GGATTTCTTTCGGCGCGATGTG
phantom reverse: TGCCTCAGTATCGAAAAGCCGT

### Puromycin assay

Groups of 10 wandering larvae or earlier time point larvae were inverted in Schneider’s media and then transferred to Eppendorf tubes containing media plus 5 μg/ml puromycin (Sigma), 6 different replicates were used for Fig 1F. The larval samples were then left to incubate for 40 minutes at room temperature. Following incubation, the inverted larvae were snap frozen for western blot analyses. For experiments on larval brains, inverted larvae were placed in ice-cold PBS after incubation with puromycin, and the brains were isolated and lysed for western blot analyses.

### Western blotting and quantification

Whole inverted larvae or isolated brains were lysed with a buffer containing 20 mM Tris-HCl (pH 8.0), 137 mM NaCl, 1 mM EDTA, 25% glycerol, 1% NP-40 and with following inhibitors: 50 mM NaF, 1 mM PMSF, 1 mM DTT, 5 mM sodium ortho vanadate (Na3VO4) and protease inhibitor cocktail (Roche cat. no. 04693124001) and phosphatase inhibitor (Roche cat. no. 04906845001), according to the manufacturer’s instruction. Protein concentrations were measured using the Bio-Rad Dc Protein Assay kit II (5000112). For each experiment, equal amounts of protein lysates for each sample (40 μg) were resolved by SDS-PAGE and electrotransferred to a nitrocellulose membrane. Blots were then briefly stained with Ponceau S to visualize total protein and then subjected to western blot analysis with specific antibodies. Protein bands were then visualized by chemiluminescence (enhanced ECL solution, Perkin Elmer). Primary antibodies used were anti-puromycin (3RH11) antibody (1:1000, Kerafast, Boston, USA, cat. no. EQ0001), anti-eIF2alpha (1:1000, AbCam #26197). Secondary antibodies were purchased from Santa Cruz 144 Biotechnology (sc-2030, 2005, 2020, 1: 10,000). Blots were quantified using ImageJ to measure anti-puromycin blot band intensities corrected for total protein measured from corresponding Ponceau S staining intensities.

### 20-Hydroxyecdysone feeding

Newly hatched *w*^*1118*^ and *rpS13/+* larvae were collected at 24 hr AEL and placed in food vials (50 larvae per vial) supplemented either with 20-hydroxyecdysone (Sigma-Aldrich CAS number 5289-74-7) or equal volume of 95% ethanol for controls. 20-hydroxyecdysone was dissolved in 95% ethanol to a final concentration of 0.3mg/ml. A minimum of four replicates was used to calculate the mean volume for each genotype.

### Immunostaining and microscopy

Drosophila larvae were fixed in 8% paraformaldehyde (Electron Microscopy Science, Hatfield, U.S.A.) in PBS at room temperature for 30 min. After blocking for 2 h in 1% BSA in PBS/0.1% Triton-X 100, inverted larvae were incubated overnight in primary antibody [anti-5HT (Sigma-Aldrich AB125, 1:2000), anti-shroud antibody (gift from R.Niwa) (1:500), anti-Syt1 antibody (1:100, Developmental Studies Hybridoma Bank). Primary antibody staining was detected using Alexa Fluor 488 (Molecular Probes) goat-anti rabbit secondary antibodies (1:5000). DNA was visualized by staining with Hoechst 33258 (Invitrogen, 1:10,000). Brains with attached prothoracic glands were then dissected out and mounted on coverslips using mounting media (Vectashield). Images were captured using a Zeiss confocal microscope LSM 880 or a Zeiss Observer. Z1 microscope.

### OPP incorporation assay for translation

Puromycin incorporation was assayed using the Click-iT Plus OPP Alexa Fluor Protein Synthesis Assay Kit (Thermo Fisher Scientific, C10456 and C10457) according to manufacturer’s instructions with minor modifications, as described below. Larvae were inverted in prewarmed Schneider’s Drosophila Medium at 25°C (Gibco, Thermo Fisher Scientific, 21720024) and transferred into a 1.5 ml tube with 1 μM OPP in Schneider’s medium for 40 min. After OPP incorporation, inverted larvae were rinsed in PBS, fixed in 4% paraformaldehyde, and rinsed again in PBS before a permeabilization and blocking step. The Click-iT reaction mix was prepared according to the manufacturer’s instructions and larvae were incubated for 30 min in the dark at room temperature. The samples were then rinsed in reaction rinse buffer and stained with Hoechst 33258 (1:10,000). Brains were dissected out and mounted in slides using mounting media (Vectashield).

### Image quantifications

For bouton numbers and relative axon lengths, brains and attached prothoracic glands were dissected from *w*^*1118*^ and *rpS13/+* larvae during the wandering L3 stage and were stained with anti-5HT and anti-shroud antibodies. Confocal Z stack images were acquired and 5-HT boutons and axon lengths of neurons that overlapped the PG (based on anti-shroud staining) were counted and measured using ImageJ2 (Version 2.3.0). The values for each individual brain were recorded and averages for each genotype were calculated. For OPP signal intensity, the mean signal intensity was measured in Fiji ImageJ2 (Version 2.3.0) for the specified genotypes within the 5-HT cell outlines. For quantification of Syt1 staining, syt1 signal intensity within the neuropil region for each brain sample was measured using Fiji ImageJ2 (Version 2.3.0).

### Statistics

Data were analyzed by Students t-test, Mann-Whitney U test, or one- or two-way ANOVA, as indicated withing the relevant figure legends. All statistical analyses and data plots were performed using Prism software (Version 9.0.1). In all figures, statistically significant differences are presented as * and indicate p<0.05.

## Supporting information

S1 Fig (to accompany Fig 1). **Development and growth rates of rpS13/+ larvae.** (A) Transcript levels of the *rpS13* heterozygotes are reduced to half the wild-type levels present in controls. mRNA was isolated from third instar wandering larvae. Total RNA was isolated and measured by qRT-PCR, n = 4 independent samples per genotype. Data are presented as +/- SEM. *p < 0.05, Student’s t-test. (B) Relative larval size of *w*^*1118*^ and *rpS13/+* animals throughout development. Larval area was measured every 24 hours after hatching until wandering and recorded as pixel area. Data are presented as box plots (25%, median and 75% values) with error bars indicating the min and max values. *p<0.05, two-way ANOVA followed by post-hoc Tukeys test, n = 22–65 larvae per genotype/timepoint. (C). Larval weight (mg) at wandering L3 stage of *w*^*1118*^ and *rpS13/+* animals. Larvae were measured in groups of 10, n = 10 independent samples per genotype. Individual data points are plotted, and the bars represent mean +/- SEM. *p < 0.05, Student’s t-test. (D) Mouth hook movements recorded in one minute of feeding for 96-hour L3 larvae of *w*^*1118*^ and *rpS13/+* animals. *w*^*1118*^ n = 20, *rpS13/+* n = 20. Individual data points are plotted, and the bars represent mean +/- SEM. *p < 0.05, Student’s t-test.
(TIF)

S2 Fig (to accompany Fig 1). **rpS13/+ larvae show no decrease in protein synthesis.** (A) Puromycin labelling of 96-hour *w*^*1118*^ and *rpS13/+* larvae. Left, Ponceau S staining showing total protein. Right, anti-puromycin immunoblot. (B) Puromycin labelling of 120-hour *w*^*1118*^ and *rpS13/+* larvae. Left, Ponceau S staining showing total protein. Right, anti-puromycin immunoblot.
(TIF)

S3 Fig (to accompany Fig 3). **Imaginal disc expression of UAS-rpS13 does not rescue the developmental delay of rpS13/+ larvae.** (A) dilp8-GFP levels in wandering third instar *w*^*1118*^ and *rpS13/+* larval imaginal discs. Scale bars, 100 μm. (B) Time to pupation in *+/+* (n = 177), *rpS13/+* (n = 84), and *rpS13/+*, *dilp8*^*EX*^*/+* (n = 100) larvae. Data are presented as +/- SEM. ns = not significant, Mann-Whitney U test. (C) Time to pupation of *+/+* and *rpS13/+* larvae with or without *UAS-rpS13* expression in imaginal cells using the *esg*^*ts*^*-Gal4* driver. Data are presented as +/- SEM. *p < 0.05, Mann-Whitney U test. n = 96 (*+/*+), 29 (*UAS-rpS13*), 99 (*rpS13/+*), 86 (*rpS13*, *UAS-rpS13/+*). (D) Time to pupation of *+/+* larvae, and *rpS13/+* larvae with or without *UAS-rpS13* expression in imaginal discs using the *nub-Gal4* driver. Data are presented as +/- SEM. *p < 0.05, Mann-Whitney U test. n = 154 (*+/*+), 127 (*rpS13/+*), 95 (*rpS13*, *UAS-rpS13/+*).
(TIF)

S4 Fig (to accompany Fig 3). **rpS13/+ larvae show no change in brain size.** Ventral nerve cord (VNC) width (μm) of brains from wandering third instar larvae of *+/+* controls (n = 12) and *rpS13/+* (n = 12) animals. Individual data points are plotted, and the bars represent mean +/- SEM. ns = not significant, Student’s t-test.
(TIF)

S5 Fig (to accompany Fig 3). **Larval brains have lower OPP incorporation than the prothoracic gland.** OPP incorporation and DNA staining of brain and prothoracic gland of *w*^*1118*^ third instar larvae. Scale bars, 100 μm.
(TIF)

S6 Fig (to accompany Fig 7). **NaChBac or Trh overexpression in 5-HT neurons does not rescue the developmental delay of rpS13/+ larvae.** (A) Time to pupation of *+/+* and *rpS13/+* larvae with or without *UAS-NaChBac* expression in serotonergic neurons using *trh-Gal4*. Data are presented as +/- SEM. *p < 0.05, Mann-Whitney U test. *+/+* (n = 154), *UAS-NaChBac* (n = 135), *rpS13/+* (n = 125), *rpS13/+*, *UAS-NaChBac*, (n = 108) animals. (D) Time to pupation of *+/+* and *rpS13/+* larvae with or without *UAS-Trh* expression in serotonergic neurons using *trh-Gal4*. *+/+* n = 93, *rpS13/+* n = 71, *UAS-trh*, *rpS13/+* n = 91. Data are presented as +/- SEM. *p < 0.05, Mann-Whitney U test.
(TIF)

S7 Fig (to accompany Fig 7). **The developmental delay of rpS24/+ larvae is partially reversed by overexpression of UAS-nsyb in 5-HT neurons.** Time to pupation of *+/+* larvae and *rpS24/+* larvae with or without *UAS-nsyb* overexpression in serotonergic neurons using *Trh-Gal4*. *+/+* n = 168, *rpS24/+* n = 105, *UAS-nsyb*, *rpS24/+* n = 124. Data are presented as +/- SEM. *p < 0.05, Mann-Whitney U test.
(TIF)
