# Supplementary figures and images for: Serotonergic neuron ribosomal proteins regulate the neuroendocrine control of Drosophila development
Source: PLoS Genet. 2022 Sep 1;18(9):e1010371. doi: 10.1371/journal.pgen.1010371 (PMC9473637; doi:10.1371/journal.pgen.1010371)

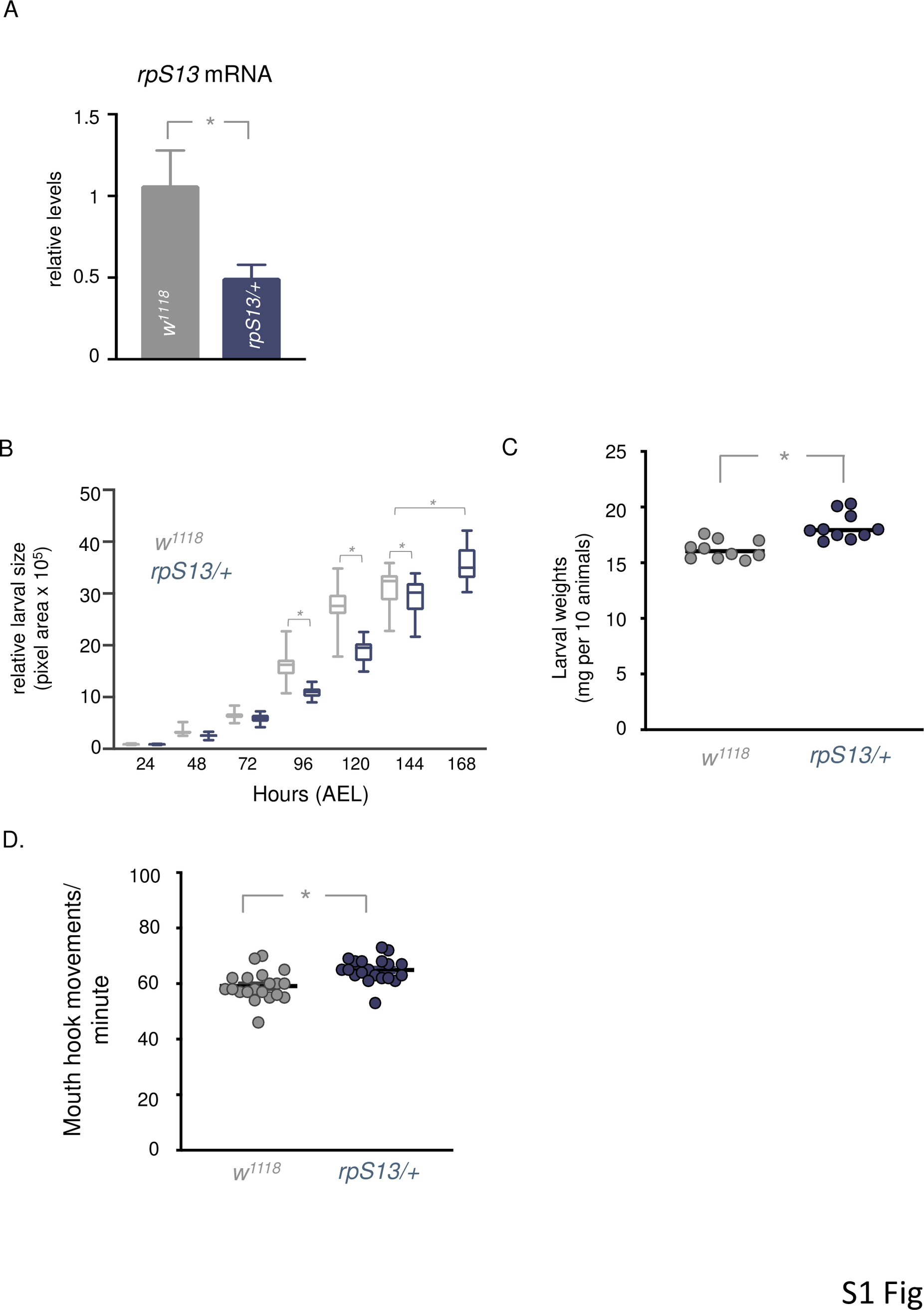

Supplement: S1 Fig — Development and growth rates of rpS13/+ larvae. (A) Transcript levels of the rpS13 heterozygotes are reduced to half the wild-type levels present in controls. mRNA was isolated from third instar wandering larvae. Total RNA was isolated and measured by qRT-PCR, n = 4 independent samples per genotype. Data are presented as +/- SEM. *p < 0.05, Student’s t-test. (B) Relative larval size of w1118 and rpS13/+ animals throughout development. Larval area was measured every 24 hours after hatching until wandering and recorded as pixel area. Data are presented as box plots (25%, median and 75% values) with error bars indicating the min and max values. *p<0.05, two-way ANOVA followed by post-hoc Tukeys test, n = 22–65 larvae per genotype/timepoint. (C). Larval weight (mg) at wandering L3 stage of w1118 and rpS13/+ animals. Larvae were measured in groups of 10, n = 10 independent samples per genotype. Individual data points are plotted, and the bars represent mean +/- SEM. *p < 0.05, Student’s t-test. (D) Mouth hook movements recorded in one minute of feeding for 96-hour L3 larvae of w1118 and rpS13/+ animals. w1118 n = 20, rpS13/+ n = 20. Individual data points are plotted, and the bars represent mean +/- SEM. *p < 0.05, Student’s t-test. (TIF) [file pgen.1010371.s001.tif]

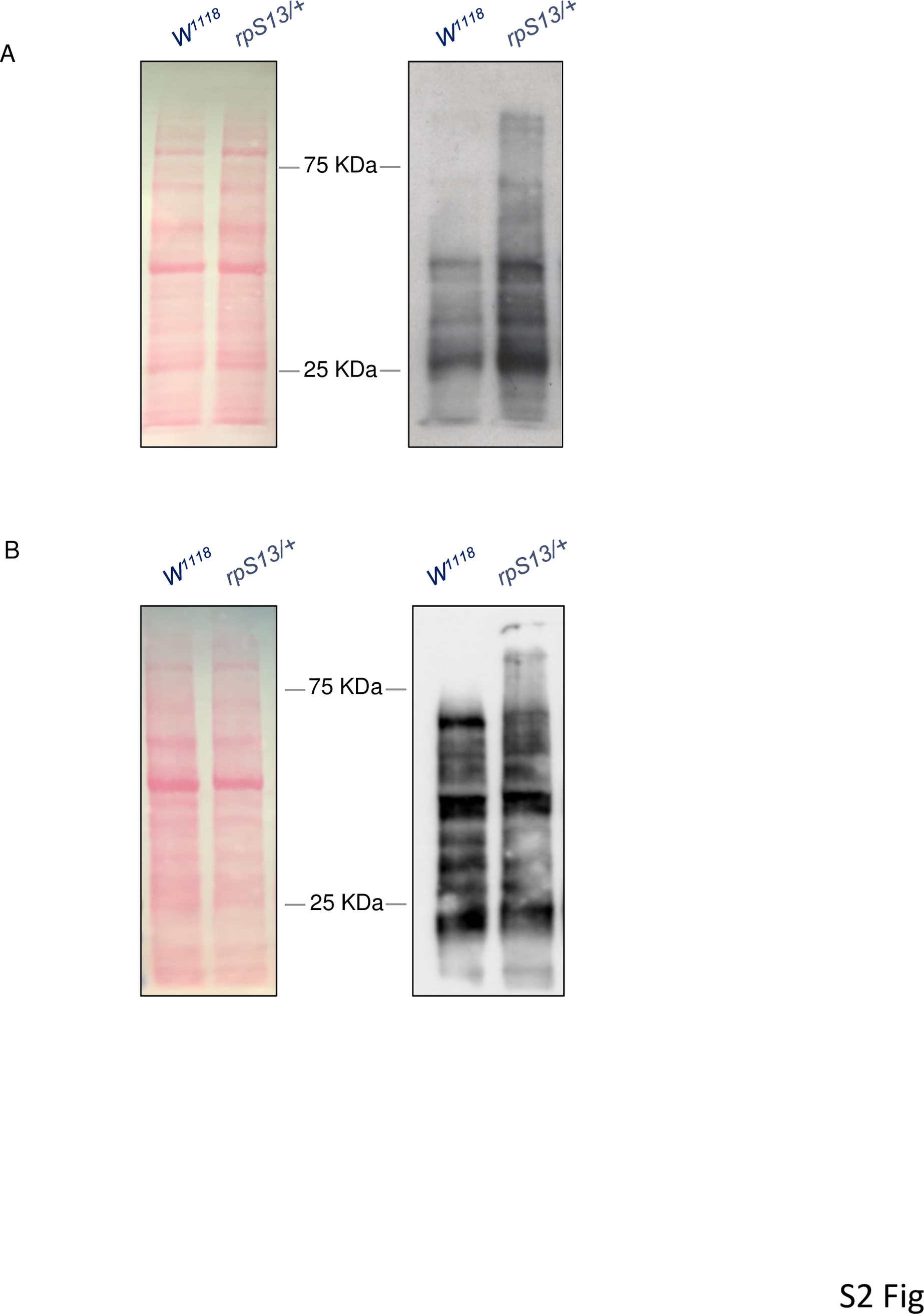

Supplement: S2 Fig — rpS13/+ larvae show no decrease in protein synthesis. (A) Puromycin labelling of 96-hour w1118 and rpS13/+ larvae. Left, Ponceau S staining showing total protein. Right, anti-puromycin immunoblot. (B) Puromycin labelling of 120-hour w1118 and rpS13/+ larvae. Left, Ponceau S staining showing total protein. Right, anti-puromycin immunoblot. (TIF) [file pgen.1010371.s002.tif]

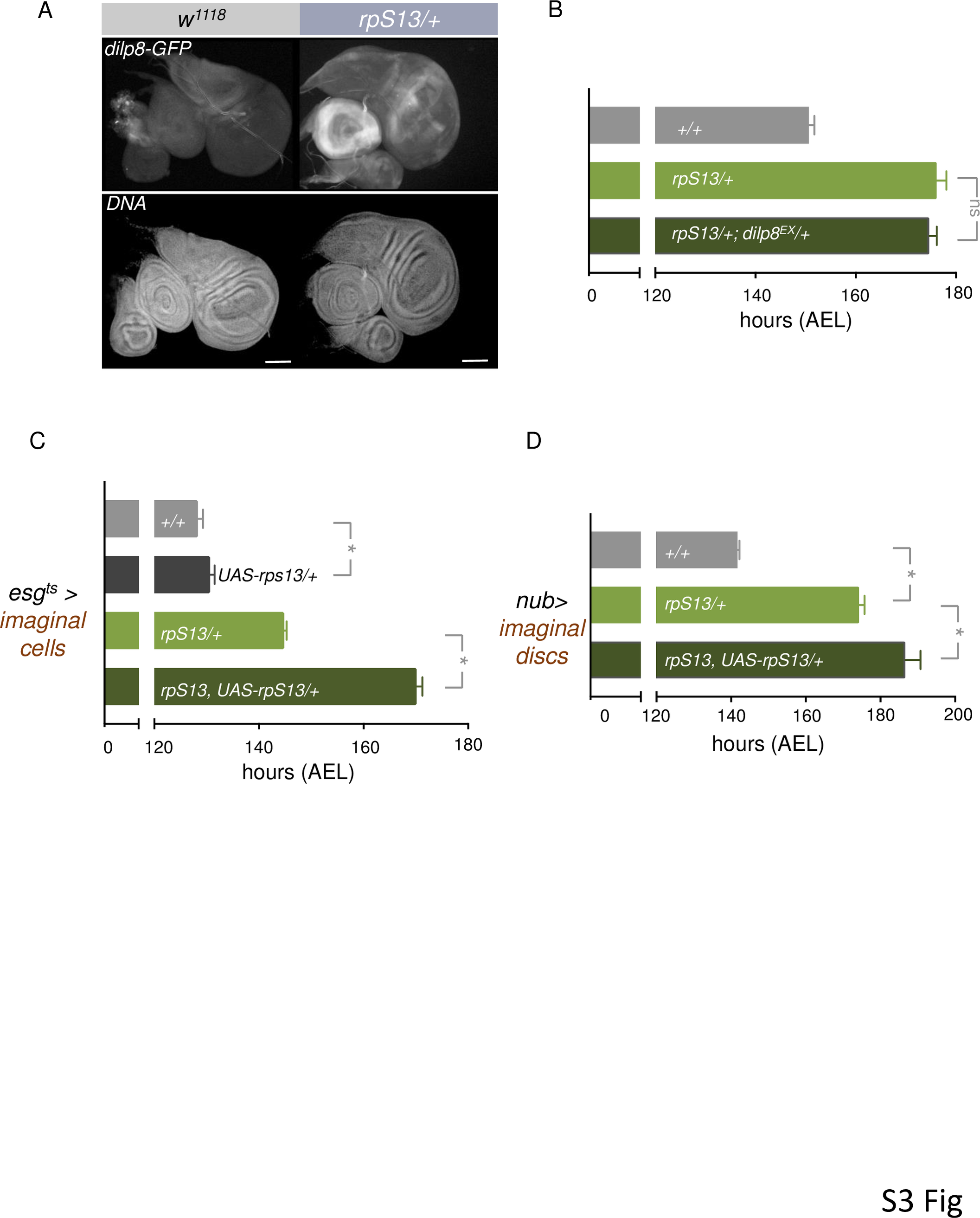

Supplement: S3 Fig — Imaginal disc expression of UAS-rpS13 does not rescue the developmental delay of rpS13/+ larvae. (A) dilp8-GFP levels in wandering third instar w1118 and rpS13/+ larval imaginal discs. Scale bars, 100 μm. (B) Time to pupation in +/+ (n = 177), rpS13/+ (n = 84), and rpS13/+, dilp8EX/+ (n = 100) larvae. Data are presented as +/- SEM. ns = not significant, Mann-Whitney U test. (C) Time to pupation of +/+ and rpS13/+ larvae with or without UAS-rpS13 expression in imaginal cells using the esgts-Gal4 driver. Data are presented as +/- SEM. *p < 0.05, Mann-Whitney U test. n = 96 (+/+), 29 (UAS-rpS13), 99 (rpS13/+), 86 (rpS13, UAS-rpS13/+). (D) Time to pupation of +/+ larvae, and rpS13/+ larvae with or without UAS-rpS13 expression in imaginal discs using the nub-Gal4 driver. Data are presented as +/- SEM. *p < 0.05, Mann-Whitney U test. n = 154 (+/+), 127 (rpS13/+), 95 (rpS13, UAS-rpS13/+). (TIF) [file pgen.1010371.s003.tif]

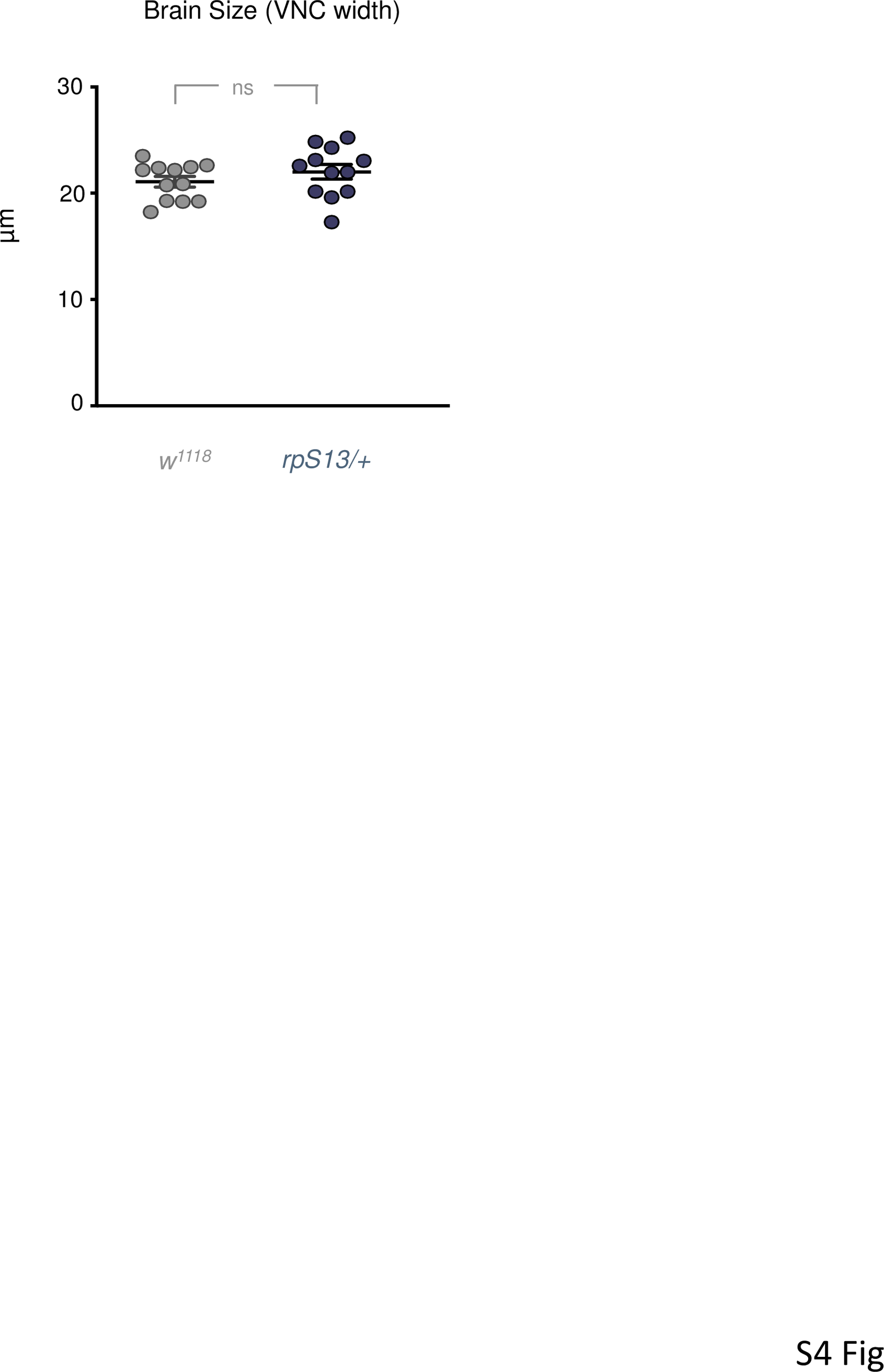

Supplement: S4 Fig — rpS13/+ larvae show no change in brain size. Ventral nerve cord (VNC) width (μm) of brains from wandering third instar larvae of +/+ controls (n = 12) and rpS13/+ (n = 12) animals. Individual data points are plotted, and the bars represent mean +/- SEM. ns = not significant, Student’s t-test. (TIF) [file pgen.1010371.s004.tif]

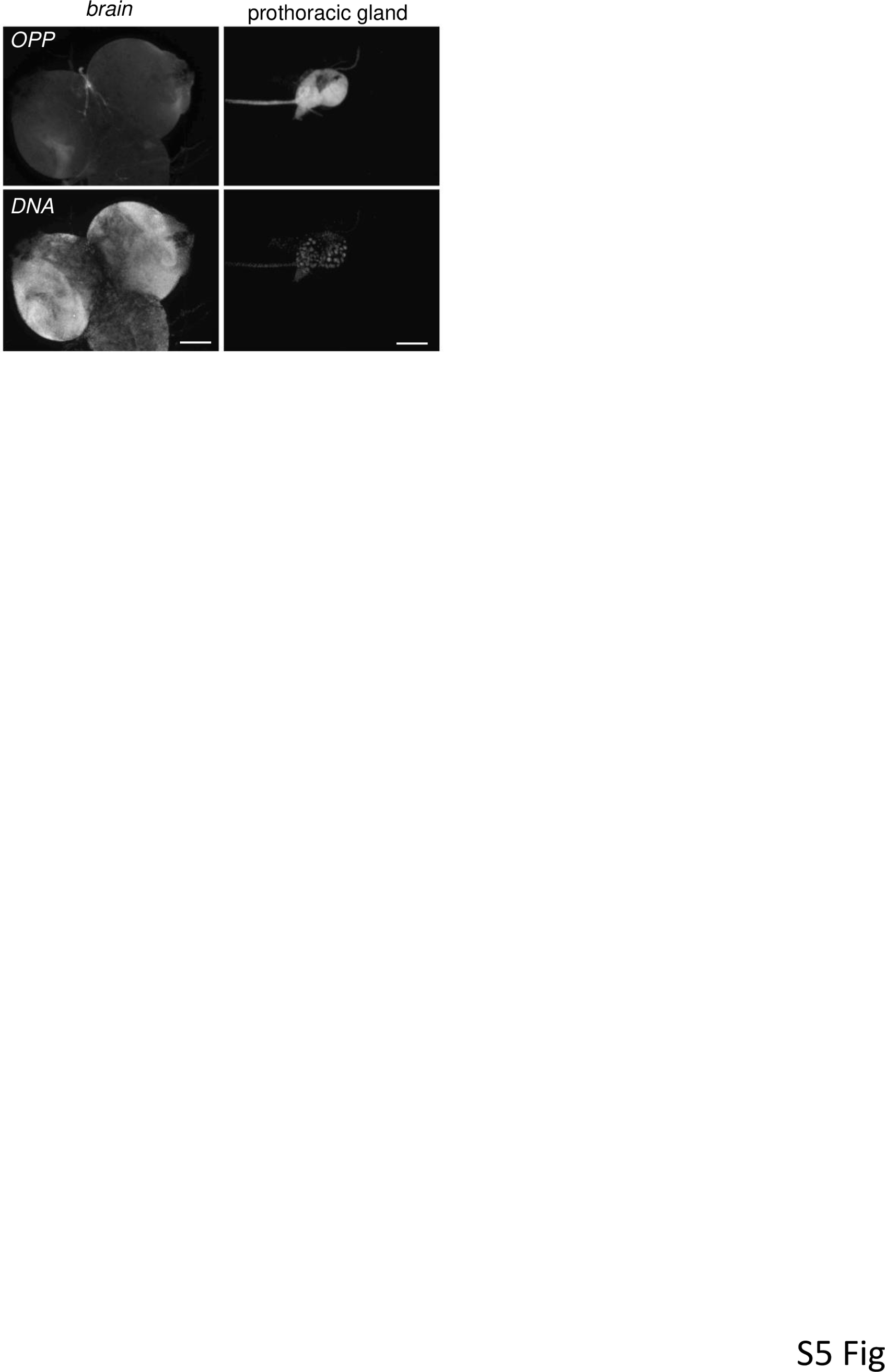

Supplement: S5 Fig — Larval brains have lower OPP incorporation than the prothoracic gland. OPP incorporation and DNA staining of brain and prothoracic gland of w1118 third instar larvae. Scale bars, 100 μm. (TIF) [file pgen.1010371.s005.tif]

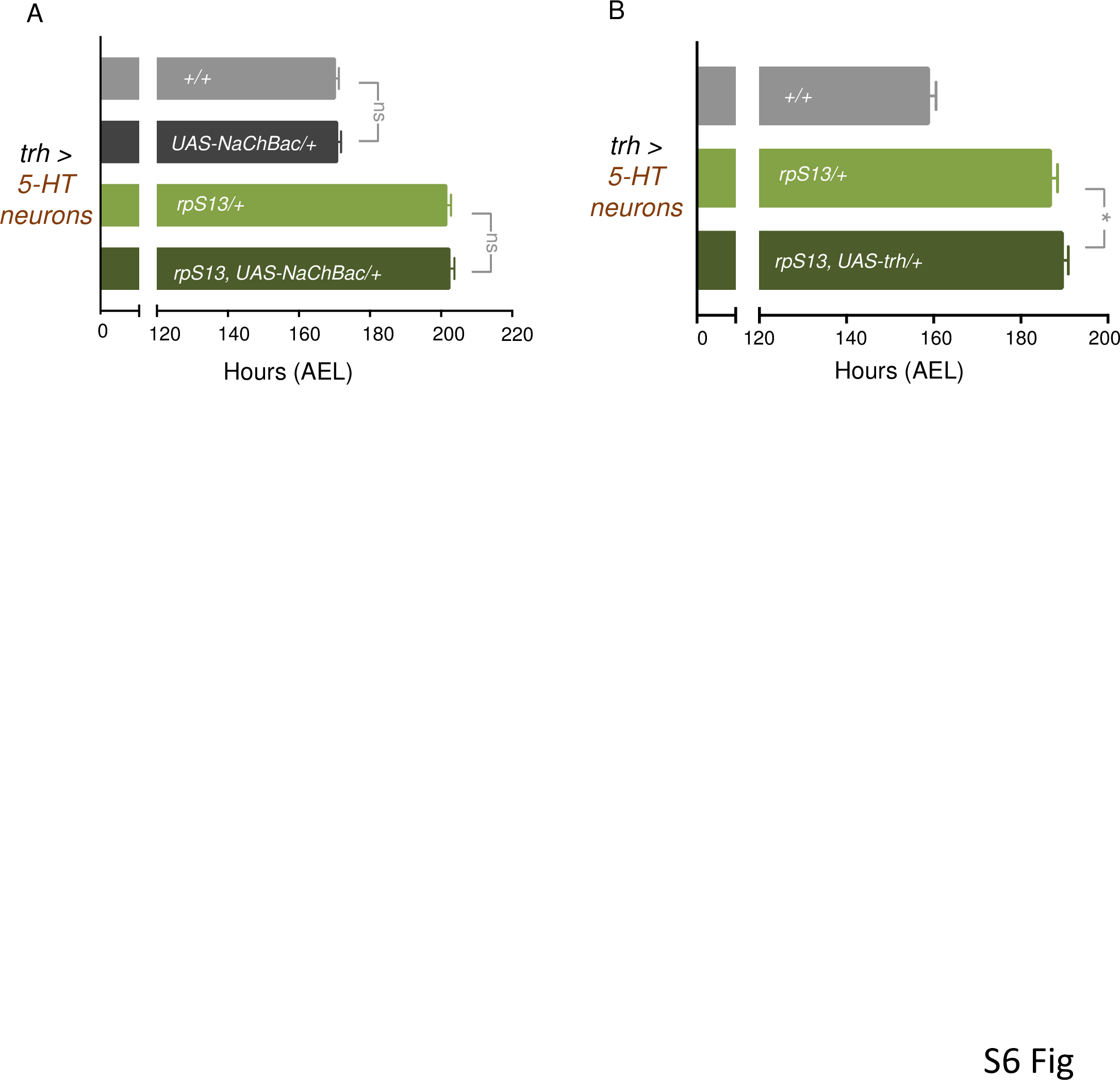

Supplement: S6 Fig — NaChBac or Trh overexpression in 5-HT neurons does not rescue the developmental delay of rpS13/+ larvae. (A) Time to pupation of +/+ and rpS13/+ larvae with or without UAS-NaChBac expression in serotonergic neurons using trh-Gal4. Data are presented as +/- SEM. *p < 0.05, Mann-Whitney U test. +/+ (n = 154), UAS-NaChBac (n = 135), rpS13/+ (n = 125), rpS13/+, UAS-NaChBac, (n = 108) animals. (D) Time to pupation of +/+ and rpS13/+ larvae with or without UAS-Trh expression in serotonergic neurons using trh-Gal4. +/+ n = 93, rpS13/+ n = 71, UAS-trh, rpS13/+ n = 91. Data are presented as +/- SEM. *p < 0.05, Mann-Whitney U test. (TIF) [file pgen.1010371.s006.tif]

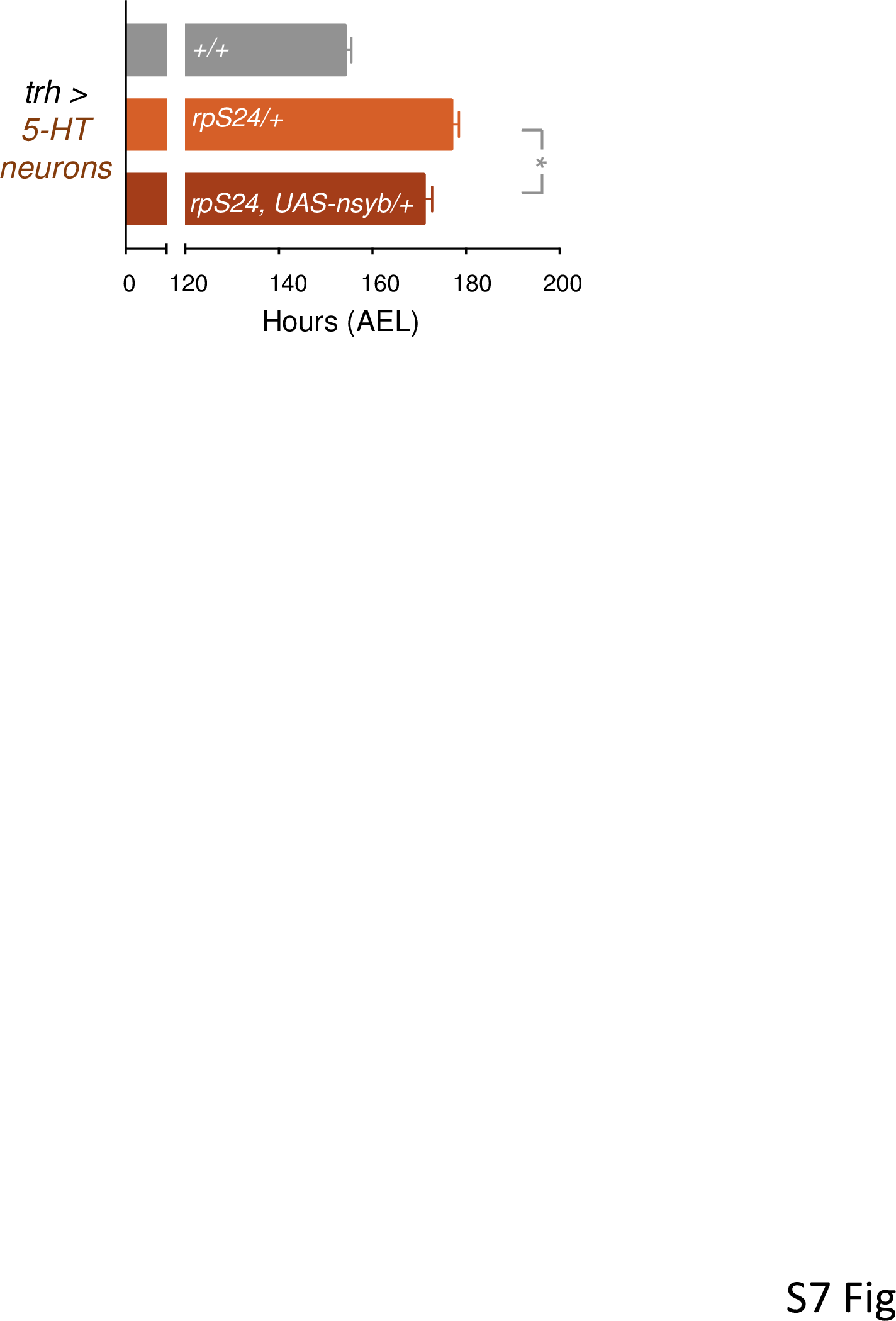

Supplement: S7 Fig — The developmental delay of rpS24/+ larvae is partially reversed by overexpression of UAS-nsyb in 5-HT neurons. Time to pupation of +/+ larvae and rpS24/+ larvae with or without UAS-nsyb overexpression in serotonergic neurons using Trh-Gal4. +/+ n = 168, rpS24/+ n = 105, UAS-nsyb, rpS24/+ n = 124. Data are presented as +/- SEM. *p < 0.05, Mann-Whitney U test. (TIF) [file pgen.1010371.s007.tif]
